# SPAG5 deficiency activates autophagy to reduce atherosclerotic plaque formation in ApoE^−/−^ mice

**DOI:** 10.1186/s12872-024-03945-5

**Published:** 2024-05-28

**Authors:** Liangyun Guo, Huijing Yuan, Huayao Zhu, Jie Zhou, Zixin Wan, Yihua Zhou

**Affiliations:** 1 Department of Ultrasound, The Second Affiliated Hospital of Nanchang University, No.1, Minde Road, Nanchang, Jiangxi, 330006 China; 2 Department of Obstetrics, Jiangxi Maternal and Child Health Hospital, No. 318 Bayi avenue, Nanchang, Jiangxi, 330006 China; 3 Department of ICU, The Second Affiliated Hospital of Nanchang University, No.1 Minde Road, Nanchang, Jiangxi, 330006, China

**Keywords:** Autophagy, Atherosclerosis, SPAG5, PI3K/Akt/mTOR

## Abstract

**Background:**

Autophagy, as a regulator of cell survival, plays an important role in atherosclerosis (AS). Sperm associated antigen 5 (SPAG5) is closely associated with the classical autophagy pathway, PI3K/Akt/mTOR signaling pathway. This work attempted to investigate whether SPAG5 can affect AS development by regulating autophagy.

**Methods:**

Human umbilical vein endothelial cells (HUVECs) were treated with oxidized-low density lipoprotein (ox-LDL) to induce cell damage. ApoE^−/−^ mice were fed a Western diet to establish an AS mouse model. Haematoxylin and eosin (H&E) staining and Oil Red O staining evaluated the pathological changes and in lipid deposition in aortic tissues. CCK-8 and flow cytometry detected cell proliferation and apoptosis. Immunohistochemistry, Enzyme linked immunosorbent assay, qRT-PCR and western blotting assessed the levels of mRNA and proteins.

**Results:**

Ox-LDL treatment elevated SPAG5 expression and the expression of autophagy-related proteins, LC3-I, LC3-II, Beclin-1, and p62, in HUVECs. GFP-LC3 dots were increased in ox-LDL-treated HUVECs and LPS-treated HUVECs. SPAG5 knockdown reversed both ox-LDL and LPS treatment-mediated inhibition of cell proliferation and promotion of apoptosis in HUVECs. SPAG5 silencing further elevated autophagy and repressed the expression of PI3K, p-Akt/Akt, and p-mTOR/mTOR in ox-LDL-treated HUVECs. 3-MA (autophagy inhibitor) treatment reversed SPAG5 silencing-mediated increase of cell proliferation and decrease of apoptosis in ox-LDL-treated HUVECs. In vivo, SPAG5 knockdown reduced atherosclerotic plaques in AS mice through activating autophagy and inhibiting PI3K/Akt/mTOR signaling pathway.

**Conclusion:**

This work demonstrated that SPAG5 knockdown alleviated AS development through activating autophagy. Thus, SPAG5 may be a potential target for AS therapy.

**Supplementary Information:**

The online version contains supplementary material available at 10.1186/s12872-024-03945-5.

## Introduction

Atherosclerosis (AS) is a chronic disease characterized by the accumulation of lipids, fiber and calcification in arteries, which is the main cause of most cardiovascular diseases (CVDs) [1, 2]. The risk of AS is no longer concentrated in western countries but involves global human health [3]. Different from the past, AS now affects more young people, and more women and individuals from different ethnic backgrounds [4]. It places an enormous burden on public health and socio-economic development. According to research it is assumed that the pathophysiological process of AS begins with the activation of vascular endothelium cells caused by abnormal tube wall shear stress [5]. Vascular endothelial activation leads to chemotaxis, recruitment, and differentiation of monocytes into macrophages [6]. Macrophages engulf oxidized-low density lipoprotein (ox-LDL) to form foam cells [7]. The accumulation of a large number of foam cells leads to the formation of lipid streaks in the intima of blood vessels. Lipid streaks further develop into lipid-rich atherosclerotic plaques encased in fibrous caps.

Autophagy is an evolutionarily conserved metabolic process that involves the degradation of aging or excess biological macromolecules and abnormal organelles within cells mediated by lysosomes [8]. It is one of the important mechanisms for maintaining the stability and survival of the intracellular environment. AS related vascular cells will start autophagy and exhibit autophagy related characteristics. Excessive autophagy leads to apoptosis of vascular cells, which in turn promotes atherogenesis [9, 10]. Multiple studies have shown that autophagy activation inhibits ox-LDL induced endothelial cell damage [11, 12]. The PI3K/Akt/mTOR signaling pathway is a classic autophagy signaling pathway that is activated in AS [13]. Inhibition of the PI3K/Akt/mTOR signaling pathway enhances ox-LDL induced autophagy and ameliorates endothelial cell damage [13, 14]. Therefore, targeting the PI3K/Akt/mTOR signaling pathway and autophagy may be feasible for the treatment of AS.

Sperm associated antigen 5 (SPAG5) is a mitotic spindle associated protein that regulates the separation of sister chromatids and plays an important role in maintaining normal cell division [15]. It has been found that SPAG5 deficiency causes centrosome replication disorder and spindle malformation [16]. Once its expression is dysregulated, it can lead to abnormal cell proliferation and even cancer transformation [17]. Silencing SPAG5 in Hela cells leads to spindle multipolarity, thereby resulting in genomic instability [18]. High expression of SPAG5 is closely associated with the progression of various cancers, such as breast cancer and hepatocellular carcinoma [19, 20]. Additionally, accumulation studies have shown that SPAG5 expression is associated with activation of PI3K/Akt/mTOR pathway [21, 22]. However, whether SPAG5 is associated with the progression of AS is unclear.

In this work, we speculated that SPAG5 may affect the progression of AS by regulating autophagy and PI3K/AKT/mTOR signaling pathway. This article may provide new therapeutic targets for the treatment of AS.

## Materials and methods

### Cell culture and treatment

Human umbilical vein endothelial cells (HUVECs) were obtained from the American Type Culture Collection (ATCC, Manassas, VA, USA) and cultured in high-glucose Dulbecco’s modified Eagle’s medium (DMEM; Solarbio Science & Technology, Beijing, China), supplemented with 10% fetal bovine serum (FBS; Solarbio Science & Technology) and 1% penicillin-streptomycin (Sangon Biotech, Shanghai, China) in a humidified 5% CO_2_ incubator at 37 °C. In vitro, the AS model was established by incubation of HUVECs with 100 µg/mL ox-LDL (Sigma-Aldrich, St. Louis, MO, USA) for 24 h. HUVECs were treated with 1 µg/mL LPS (L8880, Solarbio, Beijing, China) for 12 h. HUVECs were cultured in 0.1% DMSO as a control. HUVECs were treated with 1 mM 3-MA (Sigma-Aldrich) for 4 h.

### Cell transfection

Small interfering RNA (siRNA) and short hairpin RNA (shRNA) specially targeting SPAG5 (si-SPAG5-1, si-SPAG5-2, si-SPAG5-3 and sh-SPAG5) were used to induce SPAG5 knockdown in vitro and in vivo. Scrambled si-NC and sh-NC were served as control. HUVECs were transfected with si-SPAG5 or si-NC applying Lipofectamine™ 2000 reagent (Thermo Fisher Scientific, Waltham, MA, USA). The sh-SPAG5 and sh-NC were packaged into lentiviral particles, generating LV-sh-SPAG5 and LV-sh-NC. All oligonucleotides and lentiviral particles were obtained from Genepharm (Shanghai, China).

### Enzyme linked immunosorbent assay (ELISA)

The levels of SPAG5 in the supernatant of HUVECs were quantified using ELISA kits according to the manufacturer’s instructions. The Human SPAG5 ELISA Kit was purchased from Finetest (Wuhan, China).

### CCK-8 assay

Cell viability was measured by cell counting kit-8 (CCK-8, Thermo Fisher Scientific). HUVECs were seeded into 96-well plates at a density of 2 × 10^4^ cells/mL. CCK-8 reagent (10 µL) was added to each well for 1 h of incubation at 37℃. Finally, the absorbance at 450 nm of each well was measured by a microplate reader.

### Examination of GFP-LC3B punctation

HUVECs were seeded into coverslips and cultured for 24 h. Cells were transfected with pCMV-GFP-LC3 plasmids (Cell Biolabs, San Diego, CA, USA). After 48 h of incubation, cells were fixed with 4% formaldehyde for 15 min at room temperature. Nuclei were stained with DAPI for 5 min. LC3B puncta were observed under a confocal microscope (Olympus, Tokyo, Japan).

### Flow cytometry

Apoptosis of HUVECs was evaluated utilizing Annexin V-FITC Apoptosis Detection Kit (Beyotime, Shanghai, China). HUVECs were seeded on six-well plates at a density of 1 × 10^5^ cells/well. After incubation for 24 h, Cells were collected and suspended with 100 µL of binding buffer. PI and Annexin V-FITC solutions were then added to the cells. After incubation for 15 min, 400 µL of binding buffer was supplemented into the cells. The apoptosis rate of cells was assessed by using a CytoFLEX flow cytometer (Beckman Coulter, Fullerton, CA, USA).

### Animals

Eight-week-old male Apoe^−/−^ mice with C57BL/6J background and wild type C57BL/6J mice were purchased from Shanghai Model Organisms (Shanghai, China). Mice were raised under specific-pathogen-free conditions with a 12 h light-dark cycle. Apoe^−/−^ mice were randomly divided into 3 groups (*n* = 6): AS group, AS + LV-sh-NC group and AS + LV-sh-SPAG5 group. Control group (*n* = 6): C57BL/6J mice were fed a normal diet as a control. To silence SPAG5 in vivo, Apoe^−/−^ mice were injected with LV-sh-SPAG5 or LV-sh-NC via the tail vein. Apoe^−/−^ mice were fed an atherogenic Western diet (Research Diets, New Jersey, USA) for 12 weeks. All animal experiments were performed following the NIH Guide for the Care and Use of Laboratory Animals and approved by the Second affiliated hospital of Nanchang University.

### Western blotting

Total protein from HUVECs and aortic tissues were extracted by RIPA buffer containing 1 mmol/L PMSF (Beyotime) and quantified using the BCA Protein Assay Kit (Beyotime). Protein bands were separated by SDS-PAGE and then transferred to a polyvinylidene difluoride (PVDF) membrane. After blocking with 6% skim milk for 1 h, the PVDF membrane was incubated with primary antibodies, LC3-B (Abcam, Cambridge, MA, USA; 1:2000), Beclin-1 (Abcam; 1:2000), PI3K (Abcam; 1:1000), p-Akt (Proteintech, Wuhan, China; 1:1000), Akt (Proteintech; 1:2000), mTOR (Proteintech; 1:2000), p-mTOR (Proteintech; 1:2000) and β-actin (Proteintech; 1:5000) at 4 ℃ overnight. Then, the membrane was incubated with horseradish peroxidase-conjugated secondary antibody (Proteintech; 1:5000) for 1 h at room temperature. Finally, enhanced chemiluminescence (Beyotime) was used to visualize the immunoblot bands.

### Haematoxylin and eosin (H&E) staining

The collected aortic tissues were fixed in 4% formalin for several days and dehydrated with a series of ethanol solutions of increasing concentrations and xylene. The dehydrated tissues were embedded in paraffin wax and cut into 5 μm sections. Then, the sections were rehydrated with decreasing concentrations of ethanol. The sections were stained with hematoxylin and eosin by applying Hematoxylin and Eosin Staining Kit (Beyotime). The sections were observed with an optical microscope.

### Immunohistochemistry (IHC)

Formalin-fixed and paraffin-embedded aortic tissues were sectioned at 4 μm. Following deparaffinage and hydration, sections were subjected to antigen retrieval by microwave heating. Non-specific antigens were blocked with 1.0% normal goat serum. Primary antibodies for Beclin-1 (Abcam; 1:100), LC3B (Abcam; 1:200) and SPAG5 (Proteintech, 1:500) were incubated on the slides at 4 °C overnight. Then, the slides were incubated with a secondary antibody at room temperature. The sections were then stained with diaminobenzidine and counterstained with hematoxylin. Finally, the tissue staining was observed with an optical microscope.

### Quantitative real-time PCR (qRT-PCR)

HUVECs cells were treated with TRIzol reagent (Invitrogen, Carlsbad, CA, USA) for RNA extraction. Total RNA was reverse transcripted to cDNA utilizing PrimeScript™ RT reagent Kit (Takara, Beijing, China). The relative expression of mRNA was detected by performing a PCR reaction applying TB Green® Premix Ex Taq™ II (Takara). The results were analyzed by 2^−△△CT^.

### Oil Red O staining

Aortic tissues were immediately fixed in Tissue fixative (Servicebio, Wuhan, China), and then embedded in optimal cutting temperature (OCT) reagent (Servicebio, Wuhan, China). The aortic tissues were cut into 8 μm sections. The sections were stained with Modified Oil Red O Staining reagent (Beyotime) for 10 min. Finally, the lipid deposition of aortic tissues was observed under an optical microscope.

### Statistical analysis

The experimental results were all acquired from three independent replications Data were expressed as mean ± SD, and analyzed using GraphPad Prism9. Two-tailed Student’s t-test and one-way ANOVA were utilized to analyze the statistical difference, with a *P* value less than 0.05 as the threshold.

## Results

### Ox-LDL elevated autophagy and elevated SPAG5 expression in HUVECs

**Fig. 1:**
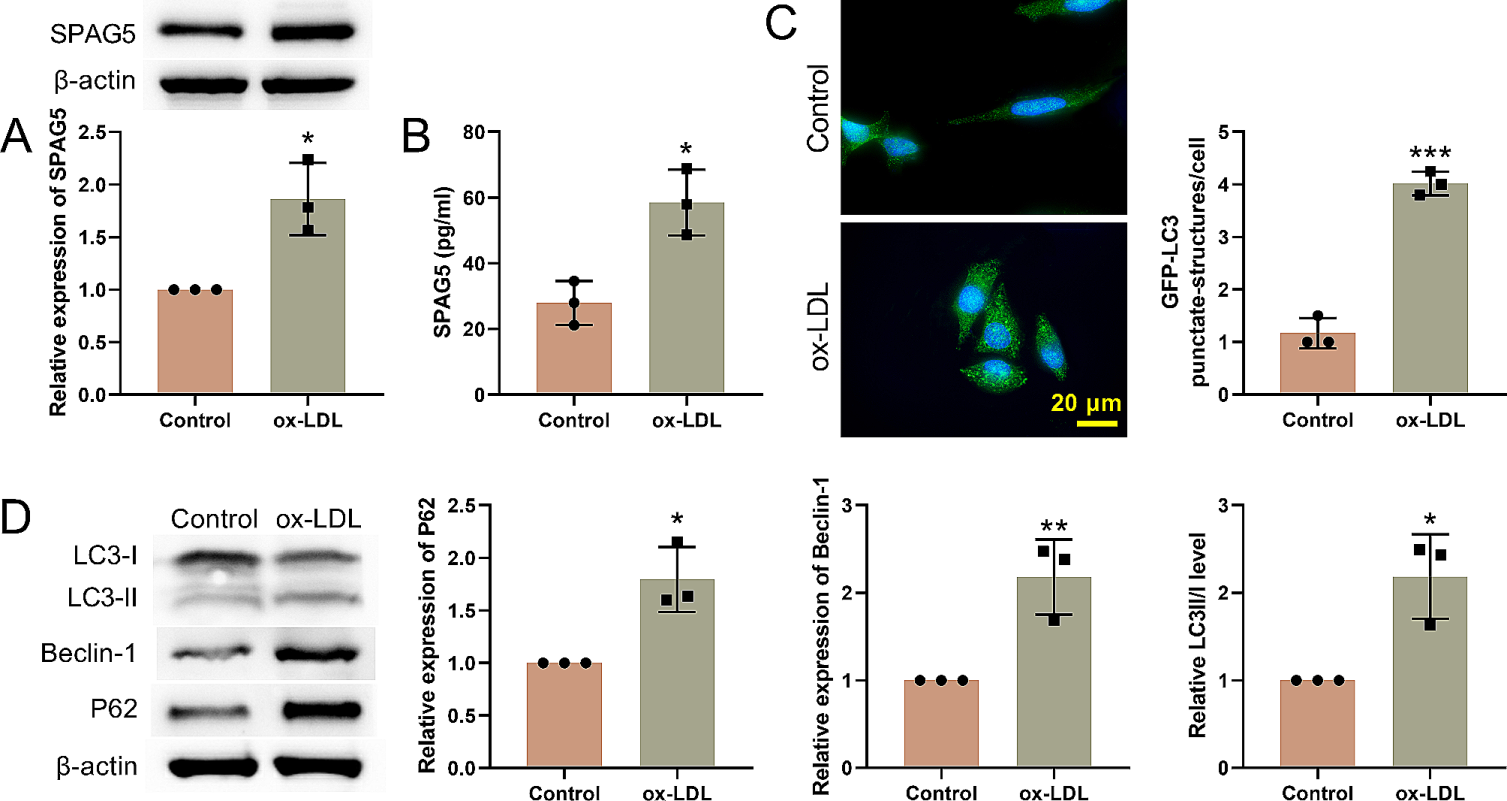
Ox-LDL treatment elevated autophagy and SPAG5 expression in HUVECs. HUVECs were treated with 100 µg/mL ox-LDL for 24 h. HUVECs were cultured in 0.1% DMSO as a control. (**A**) Western blotting examined the expression of SPAG5 in HUVECs. (**B**) ELISA assessed the levels of SPAG5 in the supernatant of HUVECs. (**C**) GFP-LC3 dots were detected. (**D**) Western blotting examined the expression of LC3-I, LC3-II, Beclin-1 and p62 in HUVECs. ^*^P < 0.05, ^**^P < 0.01, ^***^P < 0.001 vs. Control

To investigate the function role of SPAG5 in AS, we treated HUVECs with ox-LDL and examined the expression of SPAG5 in HUVECs. Up-regulation of SPAG5 was observed in HUVECs in the presence of ox-LDL, as determined by western blotting and ELISA (Fig. 1A-B). Then, the levels of GFP-LC3 punctation in HUVECs were examined, showing that ox-LDL treatment significantly increased GFP-LC3 punctation in HUVECs (Fig. 1C). The expression of autophagy-related proteins in HUVECs was assessed. As shown in Fig. 1D, the expressions of the proteins p62 and Beclin-1 were significantly increased under ox-LDL treatment. The ratio of LC3-II/LC3-I in ox-LDL treated HUVECs was markedly upregulated than that in the control group. These data indicated that ox-LDL elevated autophagy and elevated SPAG5 expression in HUVECs.

### SPAG5 silencing inhibits ox-LDL-induced damage of HUVECs

**Fig. 2:**
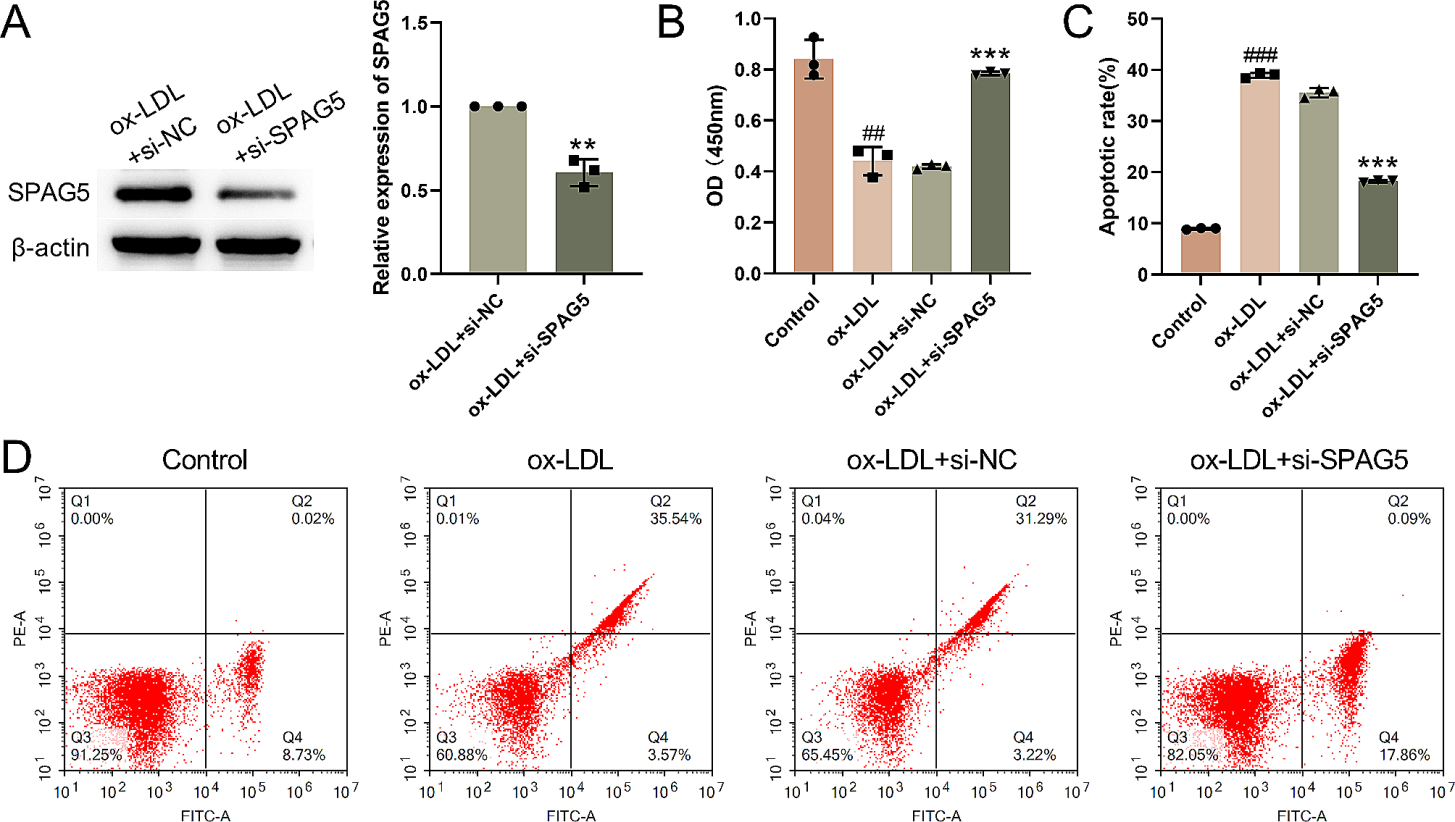
SPAG5 knockdown elevated proliferation and reduced apoptosis of ox-LDL-treated HUVECs. HUVECs were transfected with si-SPAG5 or si-NC, followed by 24 h of 100 µg/mL ox-LDL treatment. (**A**) Western blotting examined the expression of SPAG5 in HUVECs. (**B**) CCK-8 assay detected cell proliferation. (**C-D**) Flow cytometry assessed apoptosis. Q1: mechanically-dead cells; Q2 were: late apoptotic cells; Q3: alive cells; Q4: early apoptotic cells. ^##^*P* < 0.01, ^###^*P* < 0.001 vs. Control. ^**^P < 0.01, ^***^P < 0.001 vs. ox-LDL + si-NC.

To further determine the mechanism of SPAG5 in vitro, SPAG5 was silenced in HUVECs. Results of qRT-PCR and WB showed that the expression of SPAG5 was severely decreased in HUVECs in the presence of si-SPAG5-1, si-SPAG5-2, si-SPAG5-3, especially si-SPAG5-3 (Fig. S1A-B). Next, we tried to explore whether SPAG5 affects ox-LDL-induced damage of HUVECs. Results obtained from western blotting revealed that the expression of SPAG5 was severely decreased in ox-LDL treated HUVECs following transfection of si-SPAG5 (Fig. 2A). CCK-8 assay demonstrated that cell viability decreased in ox-LDL treated HUVECs. We observed that SPAG5 silencing obviously enhanced cell proliferation of ox-LDL treated HUVECs (Fig. 2B). Furthermore, cell apoptosis was detected by flow cytometry. Notably, SPAG5 down-expression impaired the ox-LDL-mediated promotion of cell apoptosis of HUVECs (Fig. 2C-D). These findings showed that SPAG5 deficiency promoted proliferation and inhibited apoptosis of ox-LDL treated HUVECs.

### SPAG5 deficiency activated autophagy and inhibited PI3K/Akt/mTOR signaling pathway in ox-LDL treated HUVECs

**Fig. 3:**
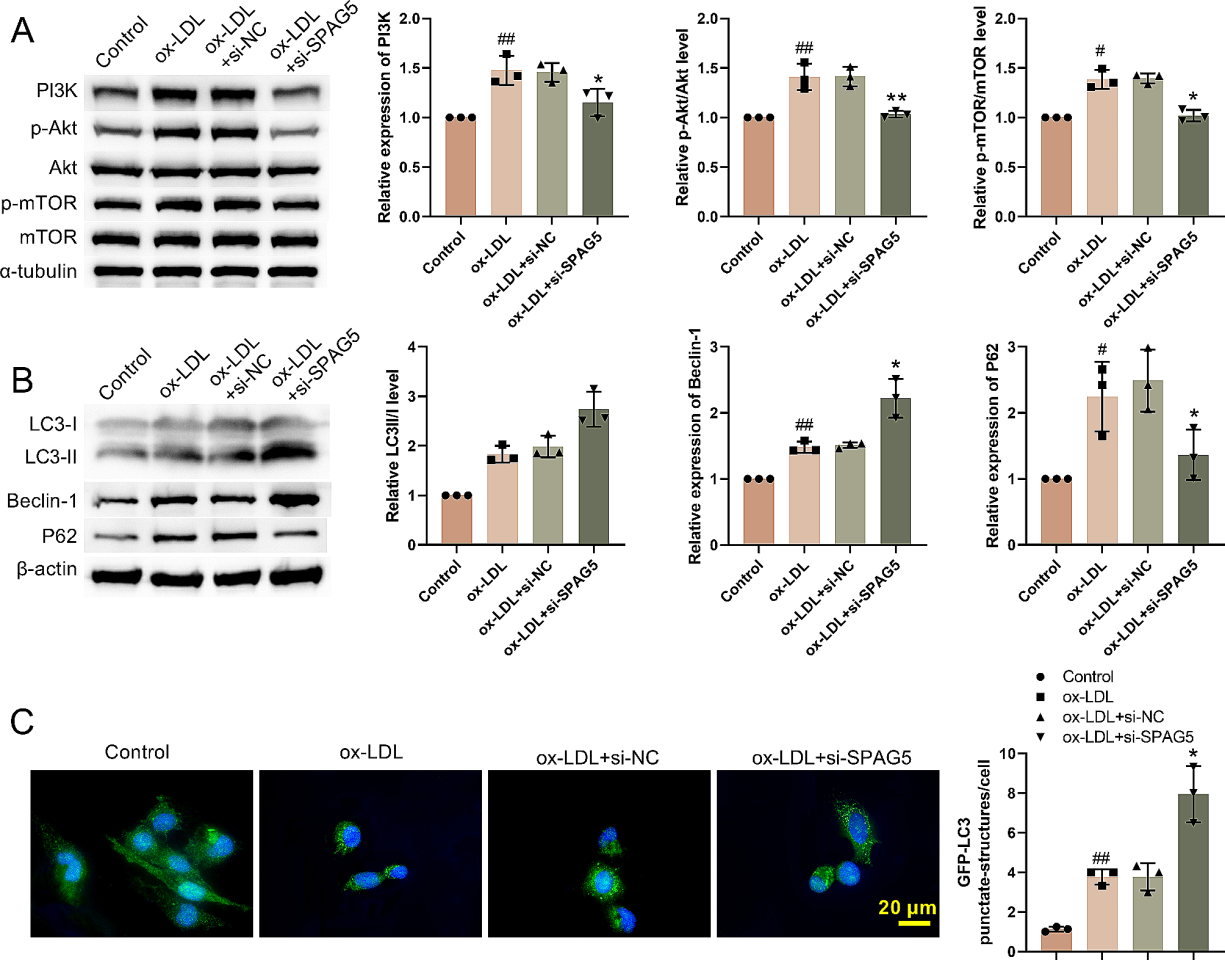
SPAG5 knockdown activated autophagy and inhibited PI3K/Akt/mTOR signaling pathway. HUVECs were transfected with si-SPAG5 or si-NC, followed by 24 h of 100 µg/mL ox-LDL treatment. (**A-B**) Western blotting examined the expression of PI3K, Akt, p-Akt, mTOR, p-mTOR, LC3-I, LC3-II, Beclin-1, and p62 in HUVECs. (**C**) GFP-LC3 dots were detected. ^#^*P* < 0.05, ^##^*P* < 0.01 vs. Control; ^*^P < 0.05, ^**^P < 0.01 vs. ox-LDL + si-NC.

To test if SPAG5 knockdown affects autophagy in ox-LDL treated HUVECs through the PI3K/Akt/mTOR signaling pathway, western blotting was utilized to detect PI3K/Akt/mTOR pathway-related proteins. As shown in Fig. 3A, the total expression of Akt and m-TOR had no change in HUVECs following ox-LDL treatment or combined with SPAG5 knockdown. Ox-LDL treatment caused an increase in the expression of PI3K, p-Akt/Akt and p-mTOR/mTOR in HUVECs, which was abolished by SPAG5 deficiency (Fig. 3A). In addition, autophagy-associated proteins LC3-II/LC3-I, Beclin-1 and p62 were notably up-regulated in HUVECs in the presence of ox-LDL. SPAG5 knockdown further elevated the expression of LC3-II/LC3-I and Beclin-1, while repressed p62 expression in ox-LDL treated HUVECs (Fig. 3B). To visualize autophagosomes by GFP-LC3, LC3 dots were examined. The levels of LC3 dots were increased in ox-LDL treated HUVECs, which was further elevated by SPAG5 silencing (Fig. 3C). Then, we used another reagent, LPS, to induce endothelial cell damage, and test the influence of SPAG5 deficiency on autophagy of HUVECs. Western blotting was utilized to detect autophagy-associated proteins. LPS treatment caused an up-regulation of LC3-II/LC3-I, Beclin-1, and p62 in HUVECs. SPAG5 knockdown greatly increased the expression levels of LC3-II/LC3-I and Beclin-1, while inhibited the expression of p62 in LPS treated HUVECs (Fig. S2A). To observe the autophagosome by GFP-LC3, LC3 dots were detected. The levels of LC3 dots were increased in LPS treated HUVECs, which was further enhanced by SPAG5 knockdown (Fig. S2B). Thus, SPAG5 deficiency promoted autophagy in ox-LDL treated HUVECs through PI3K/Akt/mTOR signaling pathway.

### SPAG5 deficiency alleviated ox-LDL-induced damage of HUVECs through activation of autophagy

**Fig. 4:**
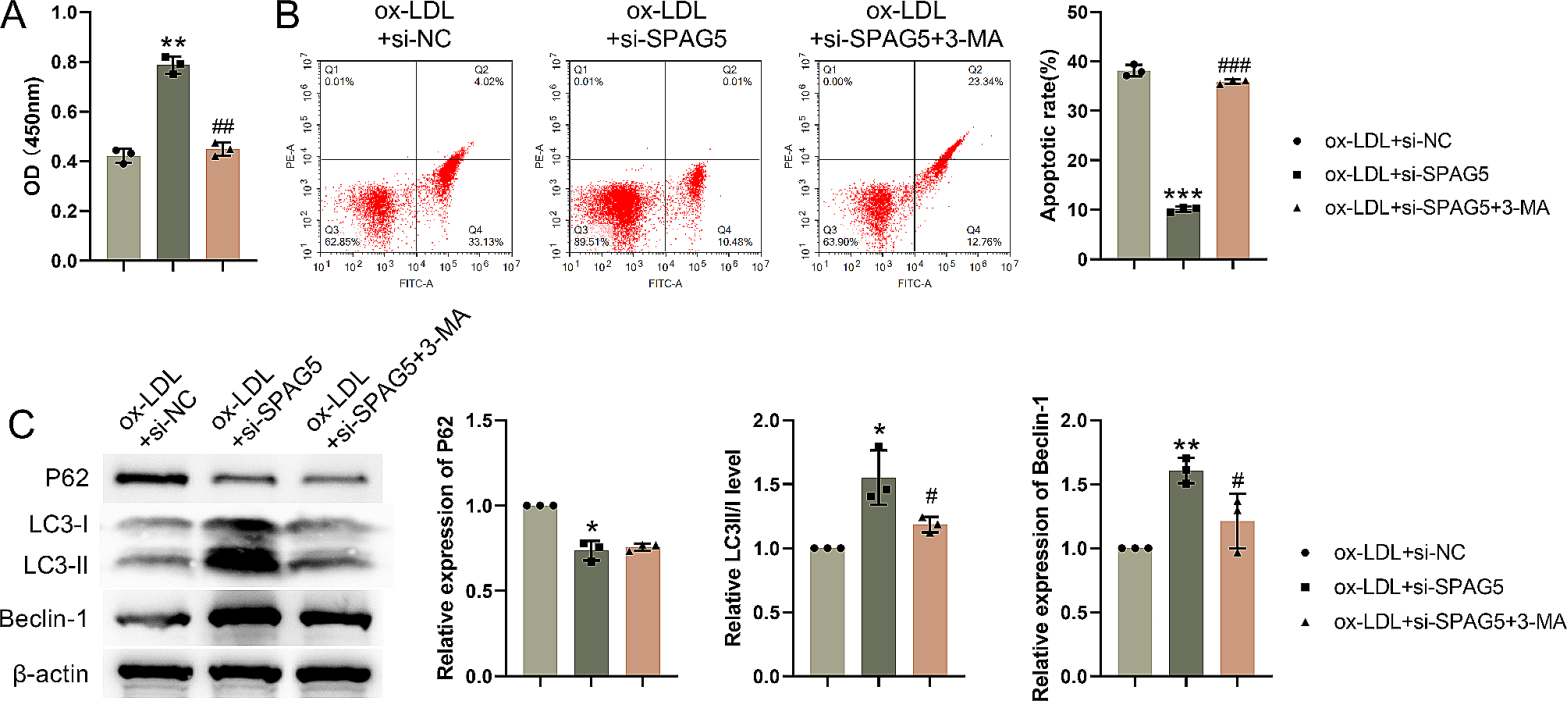
SPAG5 knockdown elevated proliferation and reduced apoptosis of ox-LDL-treated HUVECs by activating autophagy. HUVECs were transfected with si-SPAG5 or si-NC, followed by 4 h of 1 mM 3-MA treatment. (**A**) CCK-8 assay detected cell proliferation. (**B**) Flow cytometry assessed apoptosis. Q1: mechanically-dead cells; Q2 were: late apoptotic cells; Q3: alive cells; Q4: early apoptotic cells. (**C**) Western blotting examined the expression of LC3-I, LC3-II, Beclin-1 and p62 in HUVECs. ^*^P < 0.05, ^**^P < 0.01, ^***^P < 0.001 vs. ox-LDL + si-NC; ^#^*P* < 0.05, ^##^*P* < 0.01, ^###^*P* < 0.001 vs. ox-LDL + si-SPAG5.

HUVECs were treated with 3-MA, an autophagy inhibitor, to verify whether SPAG5 affects ox-LDL induced damage of HUVECs through autophagy. CCK-8 assay results showed that SPAG5 knockdown elevated cell proliferation in ox-LDL treated HUVECs. Following treatment of 3-MA, cell proliferation was decreased in HUVECs in the presence of ox-LDL and si-SPAG5 (Fig. 4A). 3-MA treatment reversed SPAG5 silencing-mediated inhibition of apoptosis in ox-LDL-treated HUVECs, as determined by flow cytometry (Fig. 4B). Then, western blotting examined the expression of autophagy-related proteins. SPAG5 knockdown repressed p62 expression and elevated the expression of LC3-II/LC3-I and Beclin-1 in ox-LDL-treated HUVECs. Importantly, there was no significant change in the level of p62 between the ox-LDL-treated HUVECs following SPAG5 knockdown or combined with 3-MA treatment. 3-MA treatment further elevated SPAG5 knockdown-mediated up-regulation of LC3-II/LC3-I and Beclin-1 in HUVECs (Fig. 4C). Therefore, SPAG5 deficiency activated autophagy to mitigate ox-LDL-induced damage of HUVECs.

### SPAG5 deficiency alleviated AS development in mice by activating autophagy

**Fig. 5:**
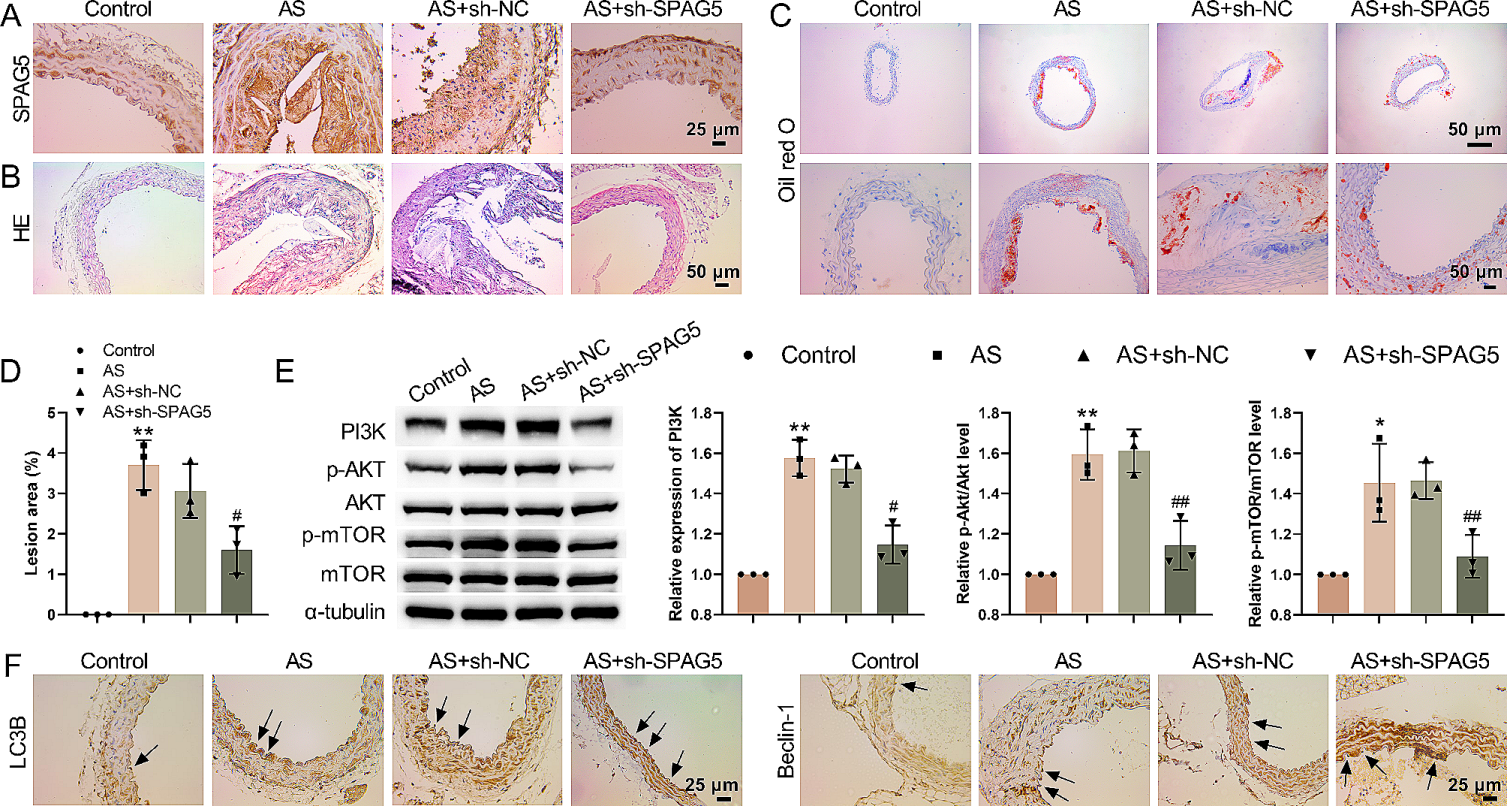
SPAG5 deficiency alleviated AS development in mice. AS mouse model was established by feeding Western diet, and then injected with LV-sh-SPAG5 or LV-sh-NC. (**A**) The expression of SPAG5 in aorta tissues was detected by immunohistochemistry. (**B, D**) HE staining detected the pathological changes of aortic tissues. (**C**) Oil Red O staining detected the lipid droplets in aorta tissues. (**E**) Western blotting examined the expression of PI3K, Akt, p-Akt, mTOR, and p-mTOR in aortic tissues. (**F**) IHC staining evaluated the expression of LC3 and Beclin-1 in aortic tissues. The black arrows indicate endothelial cells. ^*^P < 0.05, ^**^P < 0.01 vs. Control; ^#^*P* < 0.05, ^##^*P* < 0.01 vs. AS + sh-NC.

Finally, we established an AS mouse model to confirm the role of SPAG5 in vivo. IHC staining showed that the expression of SPAG5 in AS mice was significantly higher than that in the control group. Compared with the AS + sh-NC group, the expression of SPAG5 in AS + sh-SPAG5 group was significantly decreased (Fig. 5A). H&E staining evaluated the pathological changes of aortic tissues. Compared with the control group, AS mice showed protruding plaques, locally rough intima poor surface connectivity in the aorta, and disordered cell arrangement. The mice in the AS + sh-SPAG5 group showed increased surface and endothelial integrity, slightly clearer layers, and smaller plaques (Fig. 5B, D). Oil Red O staining was used to determine the lipid accumulation in AS mice. As shown in Fig. 5C, intracellular lipid droplets were significantly increased in AS mice. The levels of lipid droplets were decreased in AS mice in the presence of LV-sh-SPAG5. Moreover, we detected the effects of silencing SPAG5 on the expression of PI3K/Akt/mTOR pathway-related proteins in AS mice. Western blot results showed that the total levels of Akt and mTOR had no change in AS mice following SPAG5 knockdown. Up-regulation of PI3K, p-Akt/Akt and p-mTOR/mTOR were observed in the aortic tissues of AS mice, which was repressed by SPAG5 knockdown (Fig. 5E). Furthermore, the expression of LC3-II and Beclin-1 was also higher in aortic tissues of AS mice than that in control mice. SPAG5 deficiency further elevated the expression of LC3-II and Beclin-1 in the aortic tissues of AS mice (Fig. 5F). All these data suggested that down-regulation of SPAG5 alleviated AS development in mice by activating autophagy.

## Discussion

As an oncogene, SPAG5 has received extension attention due to its overexpression in various cancers [17]. This work first explored the mechanism of action of SPAG5 in AS. We used ox-LDL to activate autophagy and induce cell damage in HUVECs. Up-regulation of SPAG5 was observed in ox-LDL-treated HUVECs. SPAG5 knockdown elevated proliferation and inhibited apoptosis of ox-LDL-treated HUVECs, thereby alleviating ox-LDL induced cell damage in HUVECs. Moreover, 3-MA treatment reversed SPAG5 silence-mediated increase of cell proliferation and decrease of apoptosis in ox-LDL-treated HUVECs. In vivo, SPAG5 knockdown reduced atherosclerotic plaques in AS mice by inhibiting PI3K/Akt/mTOR signaling pathway and activating autophagy. Therefore, this work demonstrated that SPAG5 knockdown alleviated AS development through activating autophagy and inhibiting PI3K/Akt/mTOR signaling pathway.

SAPG5 is a member of the cancer testicular antigen family and belongs to the spindle associated protein, which is mainly involved in the dynamic regulation of the spindle during mitosis [23]. Many researchers have confirmed that SAPG5 is a driver oncogene involved in a variety of cancers. For instance, high expression of SAPG5 is observed in tumor tissues of primary hepatocellular carcinoma, which indicates a poor prognosis of hepatocellular carcinoma [24]. SPAG5 interacts with MYCBP to elevate the transcriptional activity of c-MYC, thereby accelerating DNA repair and tumor growth of triple-negative breast cancer [25]. SPAG5 silencing inhibited migration, invasion, and epithelial-mesenchymal transition of osteosarcoma cells, which is attributed to regulating the FOXM1/MMP2 axis [26]. In this work, we first explored the role of SPAG5 in AS. HUVECs were treated with ox-LDL to mimic AS conditions in vitro. At the initial stage of AS, low-density lipoprotein (LDL) accumulates within the endothelium, which is oxidized and modified to ox-LDL due to vascular endothelial damage or dysfunction [7]. Up-regulation of SPAG5 was observed in ox-LDL treated HUVECs, suggesting that SPAG5 may be associated with AS. SPAG5 knockdown reversed ox-LDL treatment-mediated inhibition of cell proliferation and promotion of apoptosis in HUVECs. Moreover, SPAG5 silencing activated autophagy and inhibited PI3K/Akt/mTOR signaling pathway. In vivo, SPAG5 knockdown reduced atherosclerotic plaques in AS mice through activating autophagy and inhibiting PI3K/Akt/mTOR signaling pathway.

Autophagy is one of the important functions for maintaining intracellular homeostasis and regulating vascular function [9]. Vascular endothelial cells are key cells involved in the formation and development of atherosclerotic plaques [27]. Different degrees of autophagy exist within vascular endothelial cells to maintain intracellular homeostasis, and the degree of autophagy affects plaque stability [28]. A previous study has confirmed that hsa_circ_0030042 regulates autophagy by sponging eIF4A3, which contributes to ameliorate plaque stability in high-fat-diet-fed ApoE^−/−^ mice [29]. In ox-LDL-treated macrophages and atherosclerotic plaques of ApoE^−/−^ mice, the function of autophagy is impaired [30]. ATG14 overexpression enhances the fusion of autophagosomes with lysosomes and inhibits ox-LDL-induced damage in macrophages. These data indicate that autophagy plays a crucial role in AS development, which is consistent with the present work. Following treatment of ox-LDL, the expression of autophagy-related proteins, LC3-I, LC3-II, Beclin-1 and p62, was up-regulated in HUVECs. SPAG5 knockdown up-regulated these autophagy-related proteins and inhibited the expression of PI3K/Akt/mTOR-related proteins in ox-LDL-treated HUVECs. PI3K/Akt/mTOR is a key signaling pathway that regulates autophagy, and then affects the development of cardiovascular diseases [31, 32]. Previous studies have reported that SPAG5 triggers PI3K/AKT signaling pathway to exert oncogenic activities in various cancers, such as hepatocellular carcinoma, and gastric cancer [20, 33]. Consistently, PI3K/Akt/mTOR signaling pathway was inactivated by SPAG5 knockdown in ox-LDL-treated HUVECs. However, further research is needed to determine whether SPAG5 knockdown affects the autophagy of endothelial cells through PI3K/Akt/mTOR signaling pathway. Therefore, these data suggested that SPAG5 affected AS development by activating autophagy and inhibiting PI3K/Akt/mTOR signaling pathway.

In conclusion, this work demonstrated that SPAG5 knockdown alleviated AS development through activating autophagy and inhibiting PI3K/Akt/mTOR signaling pathway. SPAG5 may be a potential target for AS treatment.

### Electronic supplementary material

Below is the link to the electronic supplementary material.

Supplementary Material 1

Supplementary Material 2

## Data Availability

The datasets used and/or analyzed during the current study are available from the corresponding author upon reasonable request.
