# Supplementary material for: SPAG5 deficiency activates autophagy to reduce atherosclerotic plaque formation in ApoE−/− mice
Source: BMC Cardiovasc Disord. 2024 May 28;24:275. doi: 10.1186/s12872-024-03945-5 (PMC11131316; doi:10.1186/s12872-024-03945-5)

Fig.1A-SPAG5


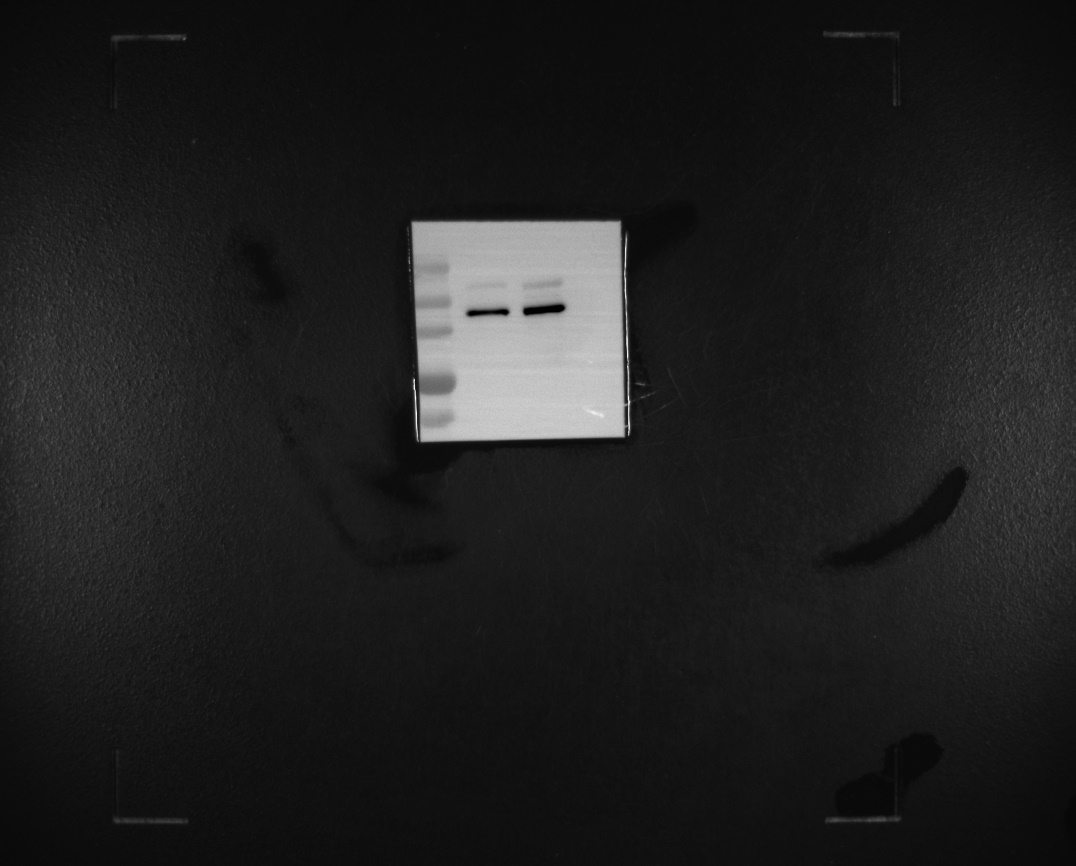


Fig.1A-β-actin


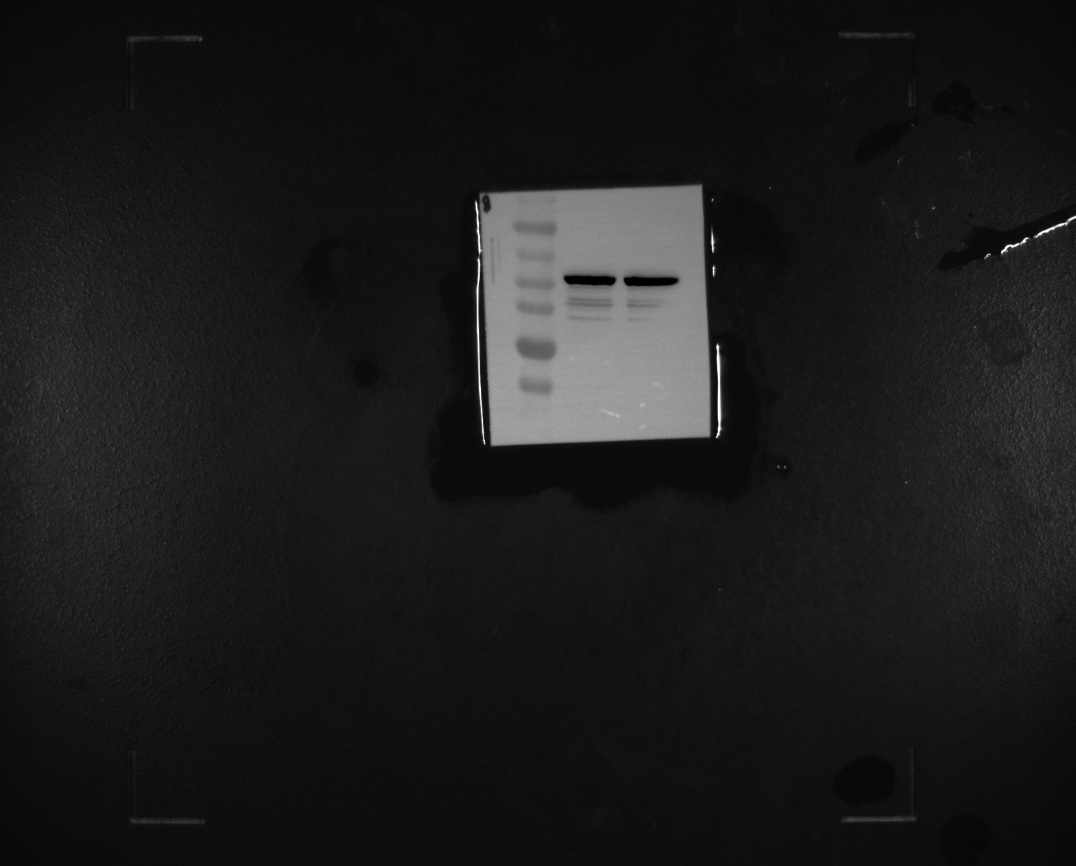


Fig.1D- LC3Ⅰ-Ⅱ


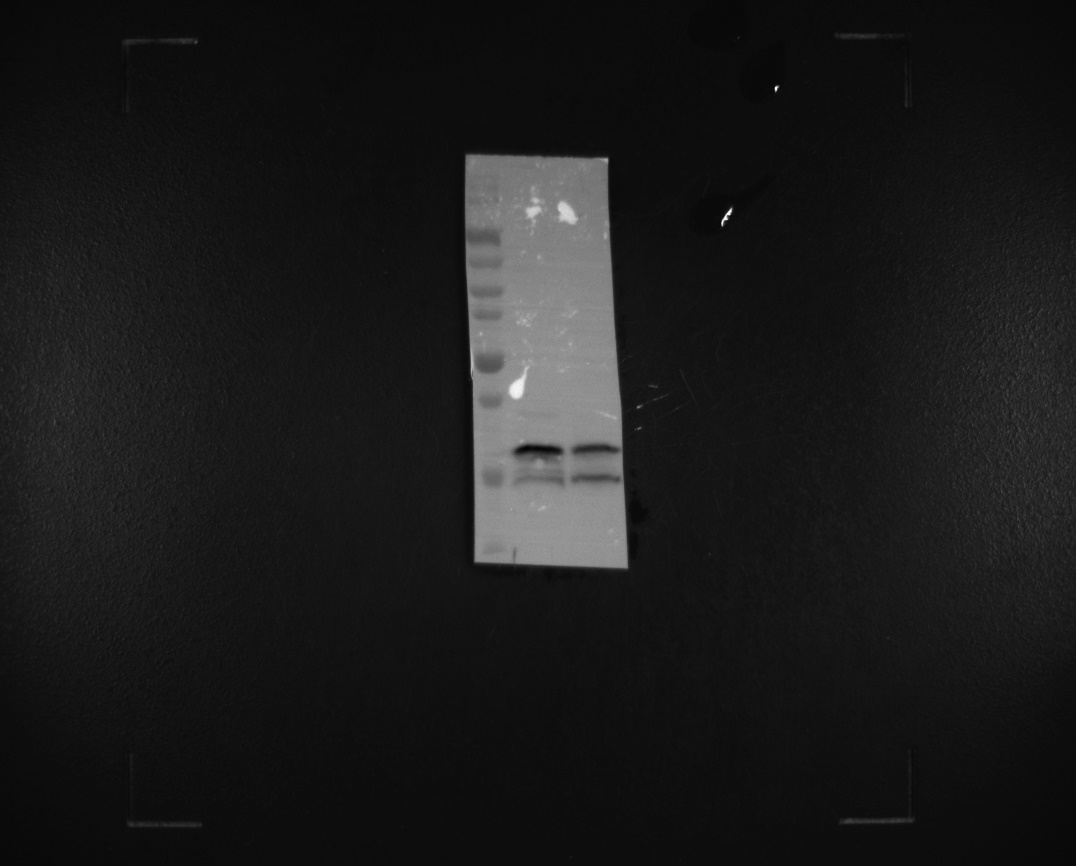


Fig.1D-Beclin-1
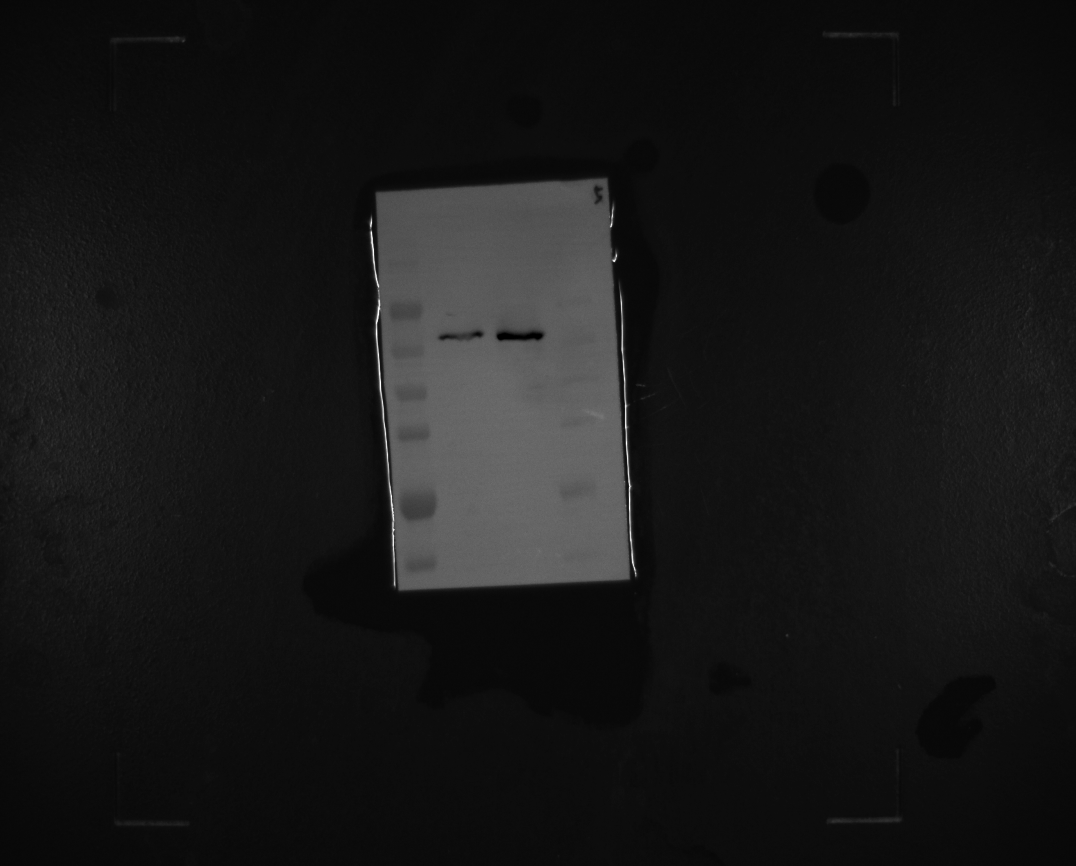


Fig.1D-P62


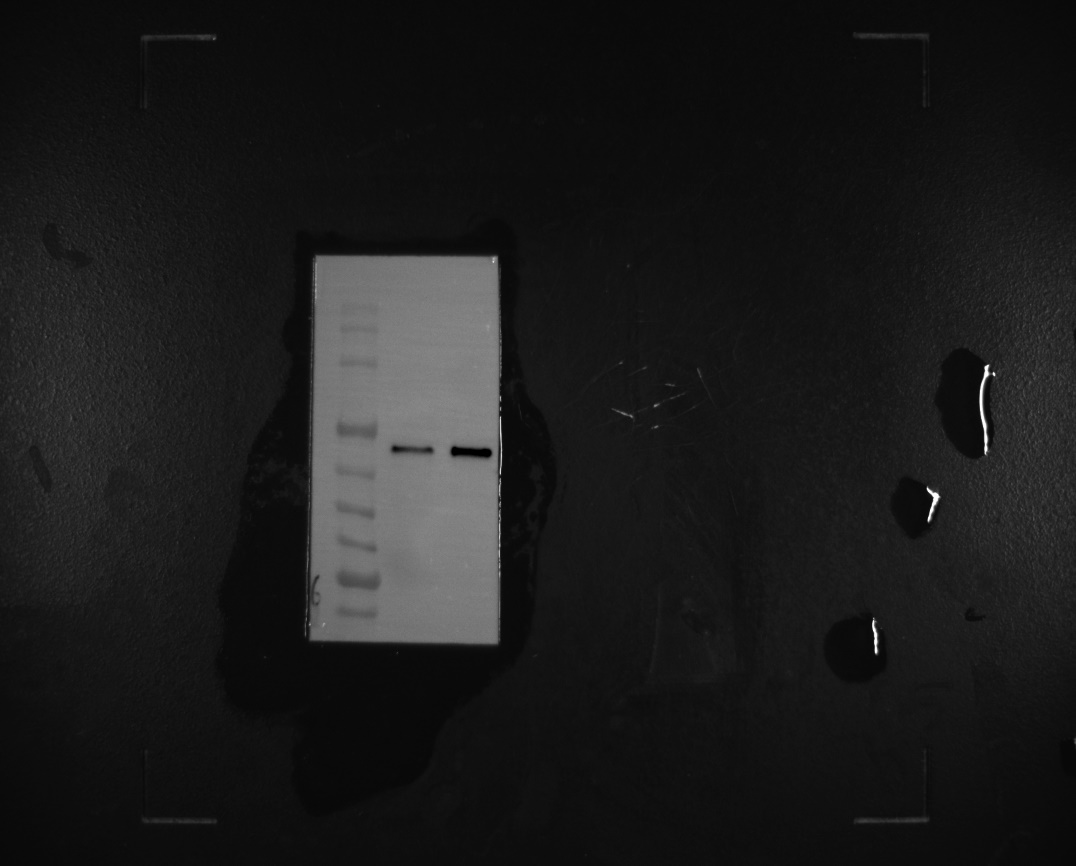


Fig.1D-β-actin


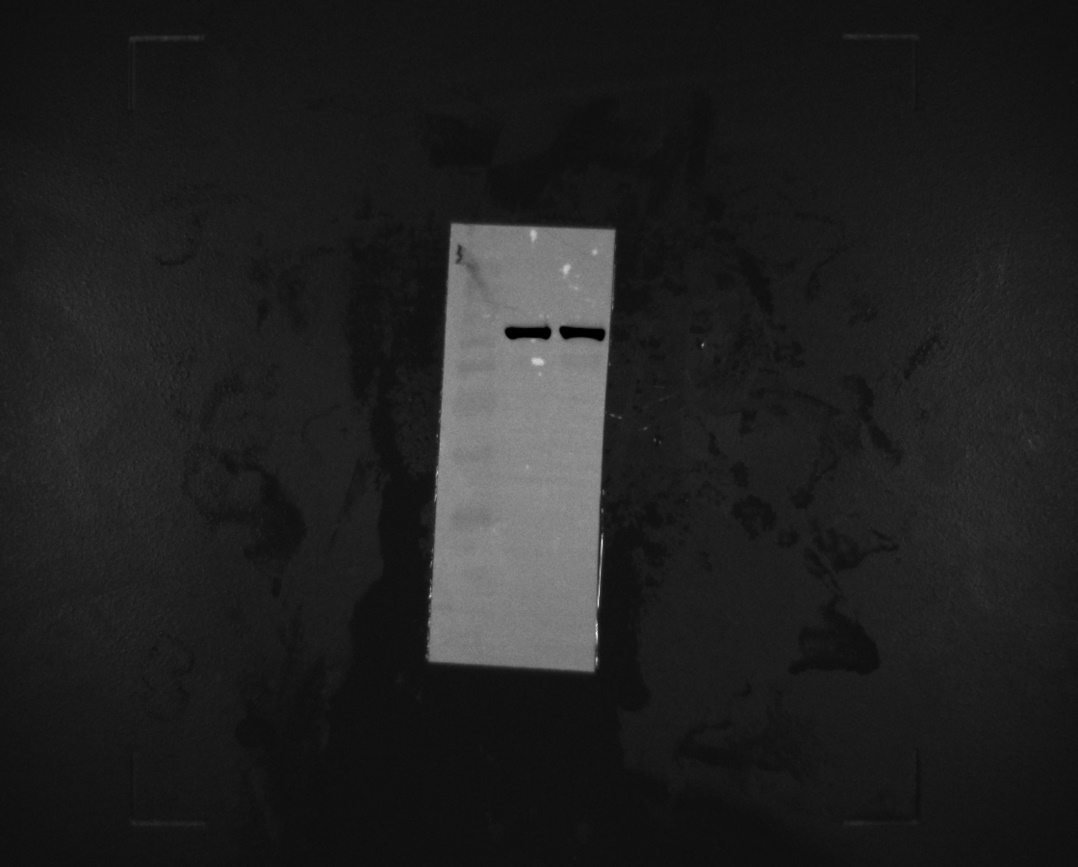


Fig.2A-SPAG5-M


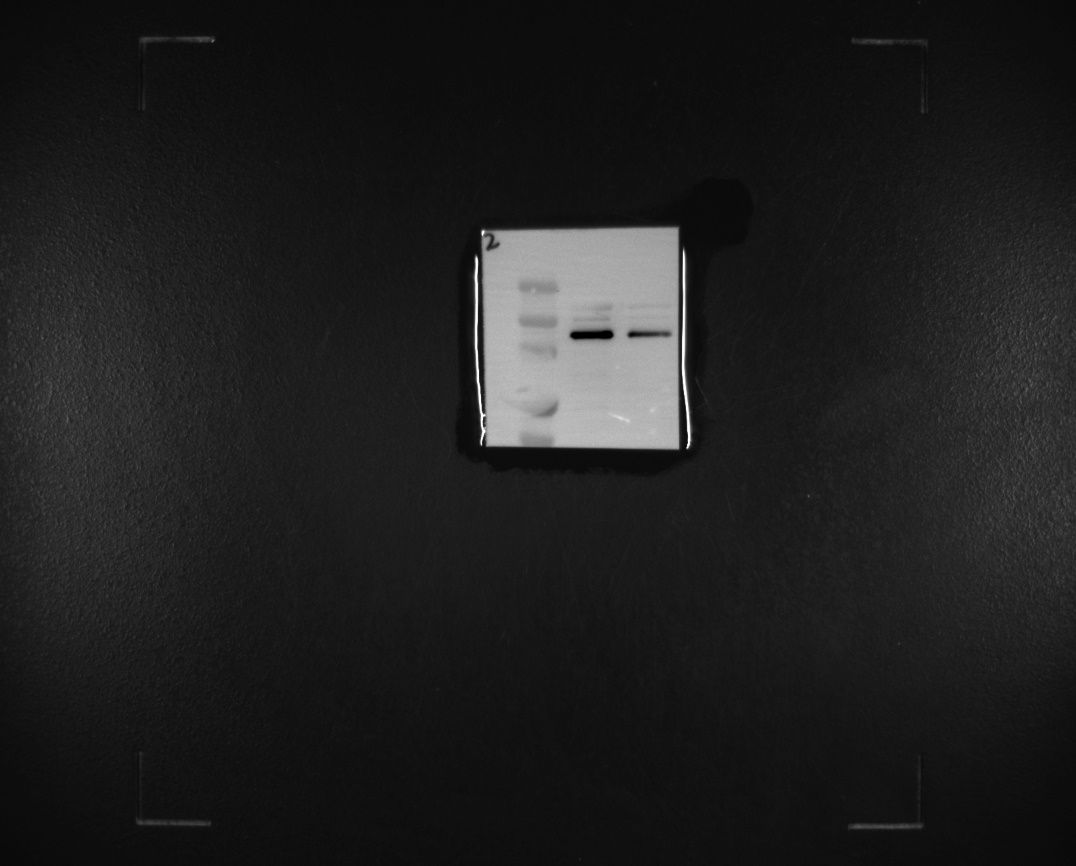


Fig.2A-β-actin


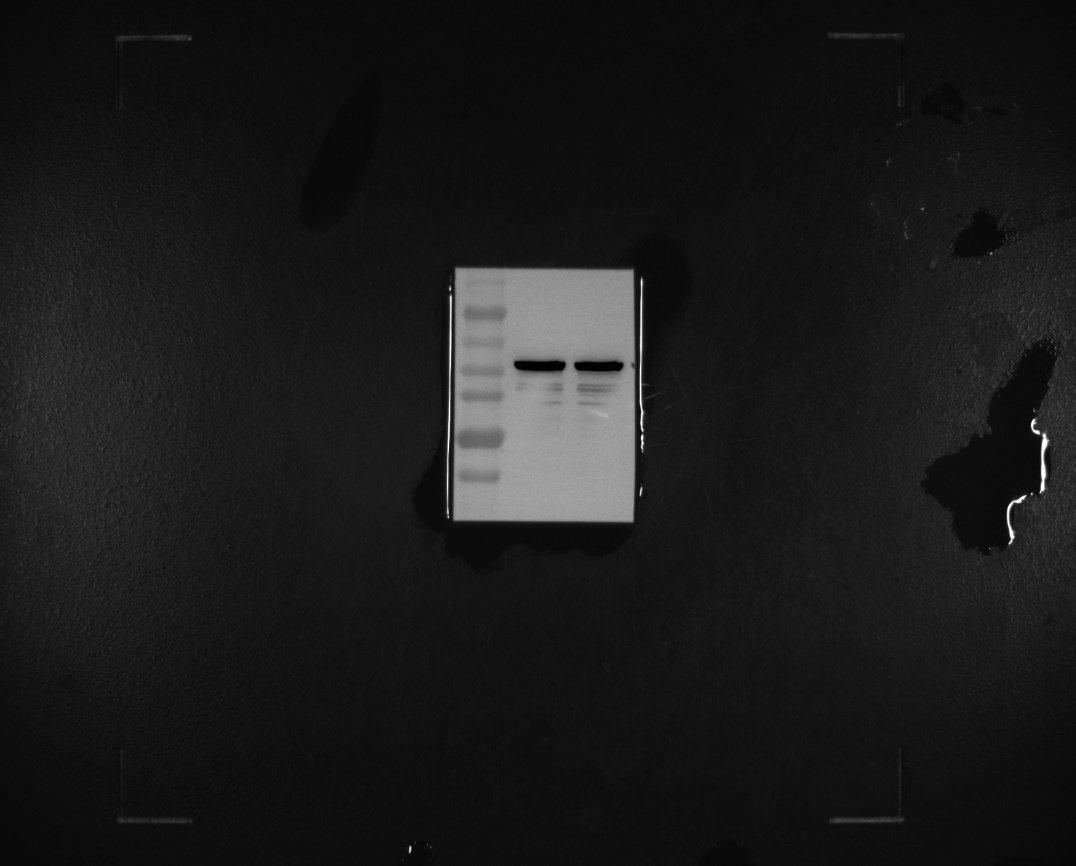


Fig.3A-PI3K


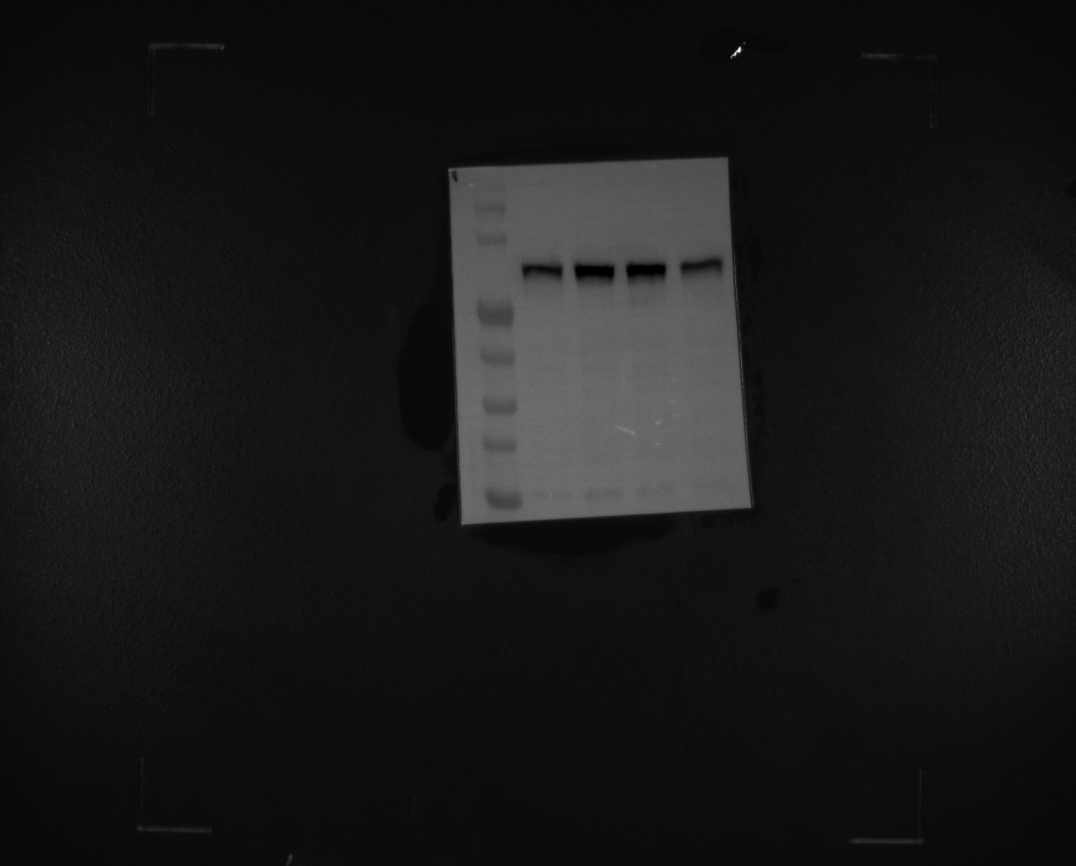


Fig.3A-p-Akt


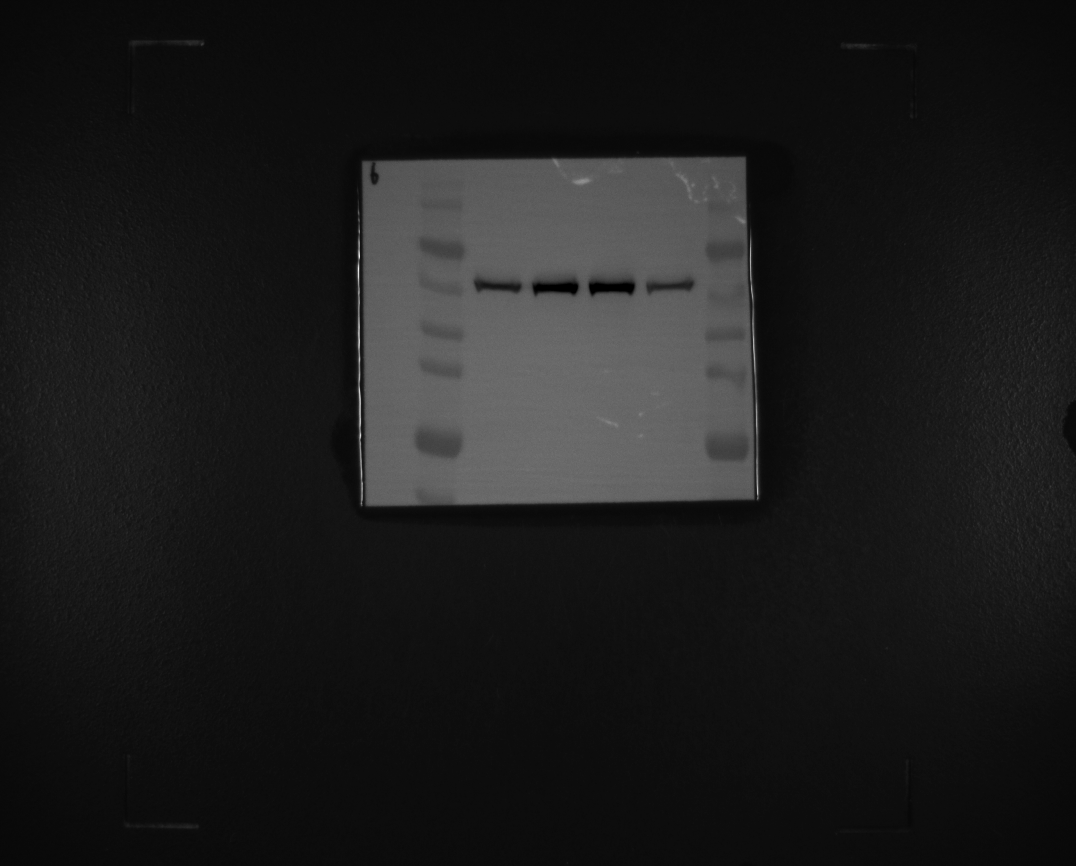


Fig.3A-Akt


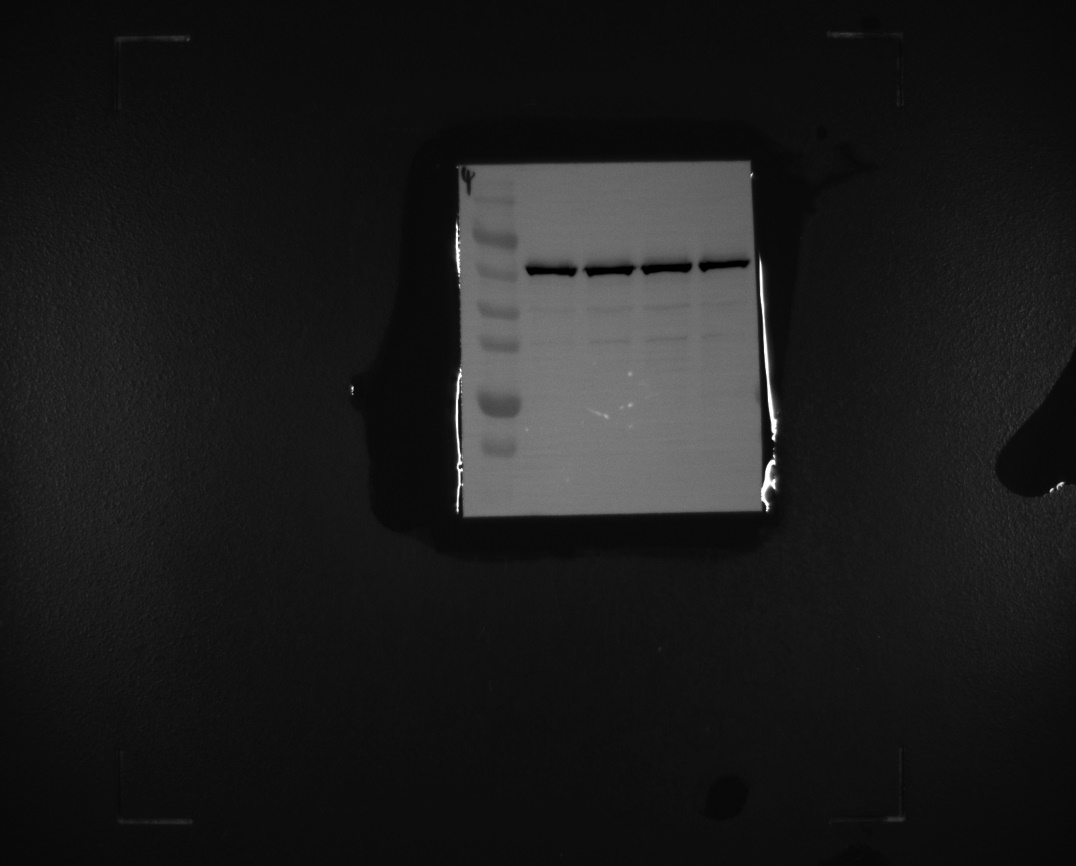


Fig.3A-p-mTOR


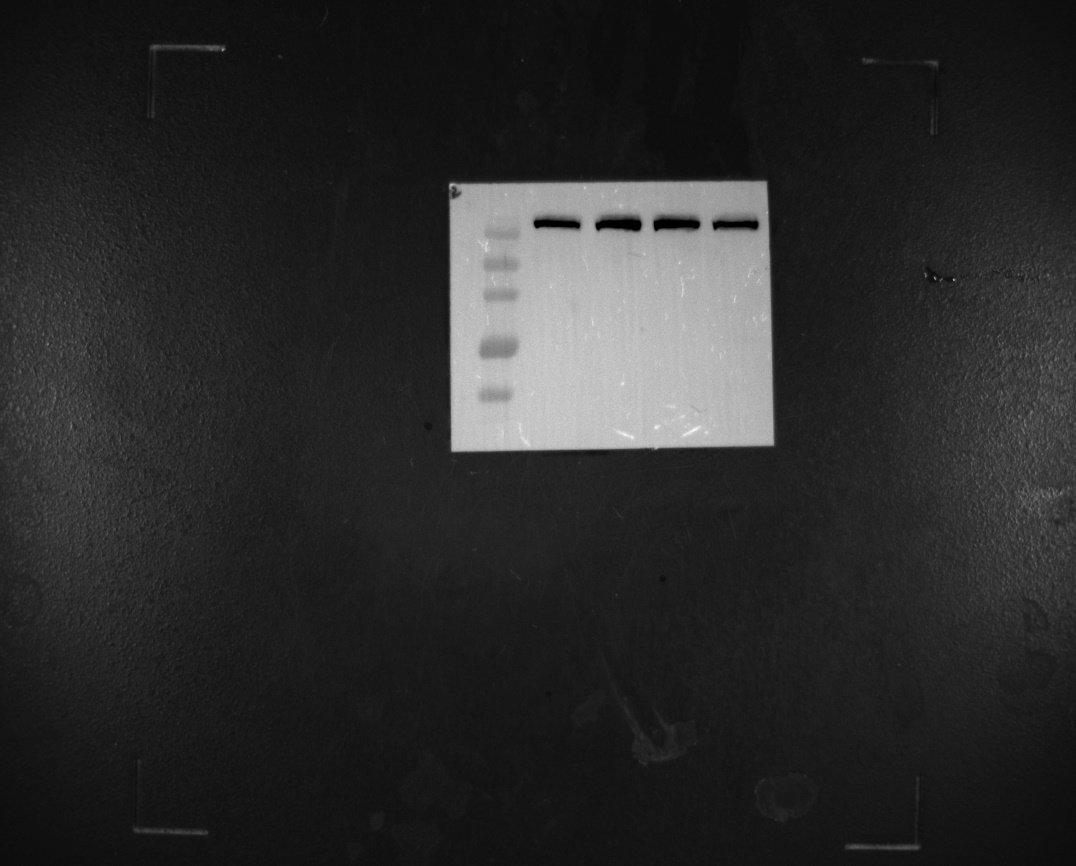


Fig.3A-mTOR


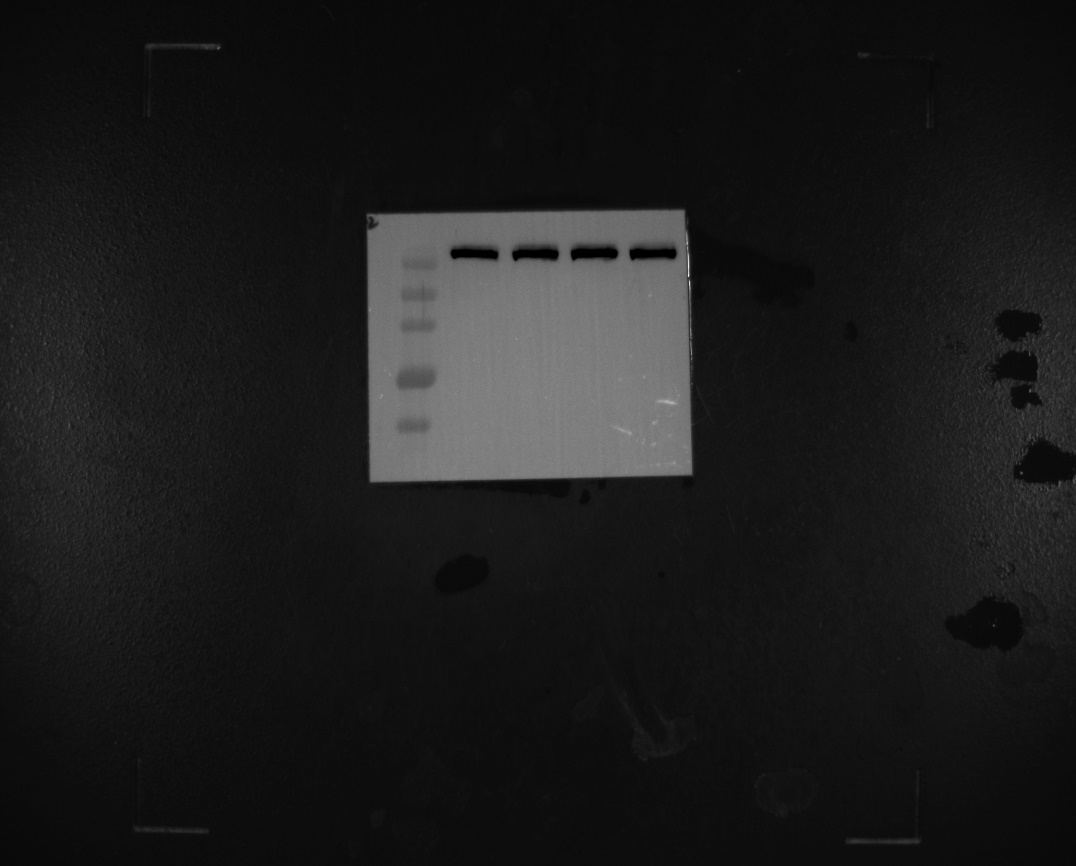


Fig.3A-α-tublin


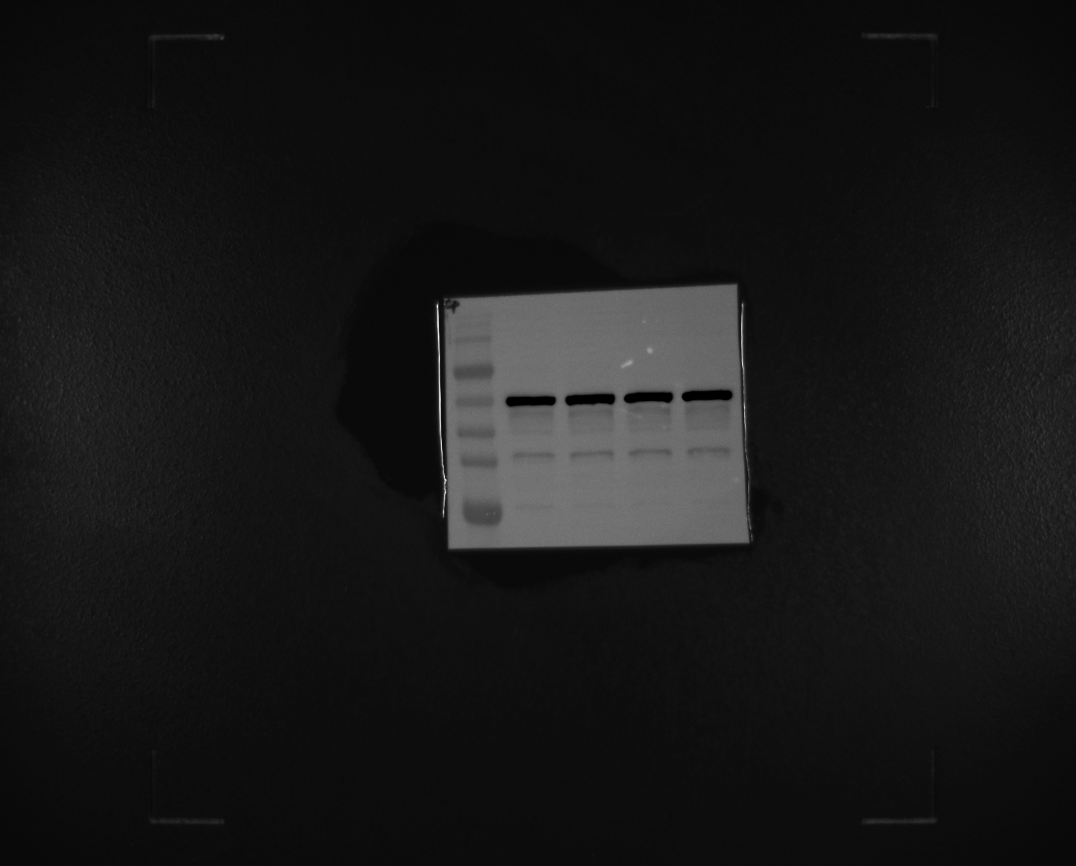


Fig.3B-LC3Ⅰ-Ⅱ


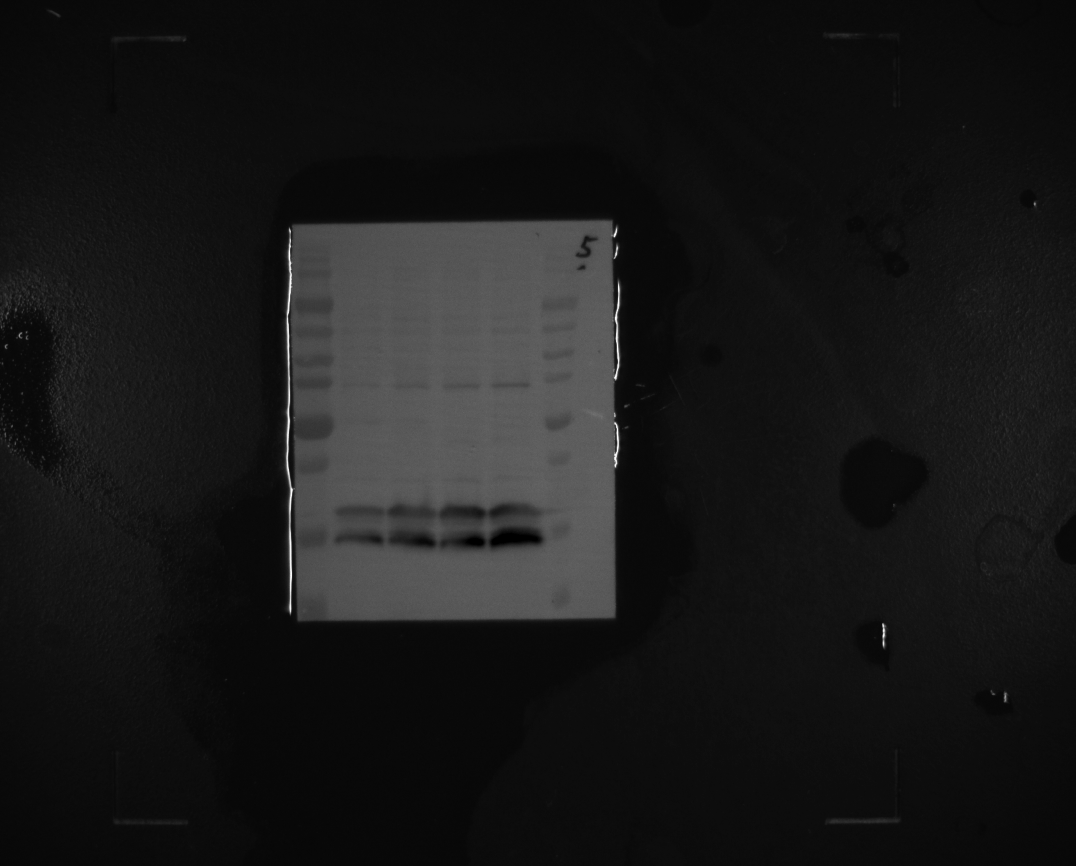


Fig.3B-Beclin-1


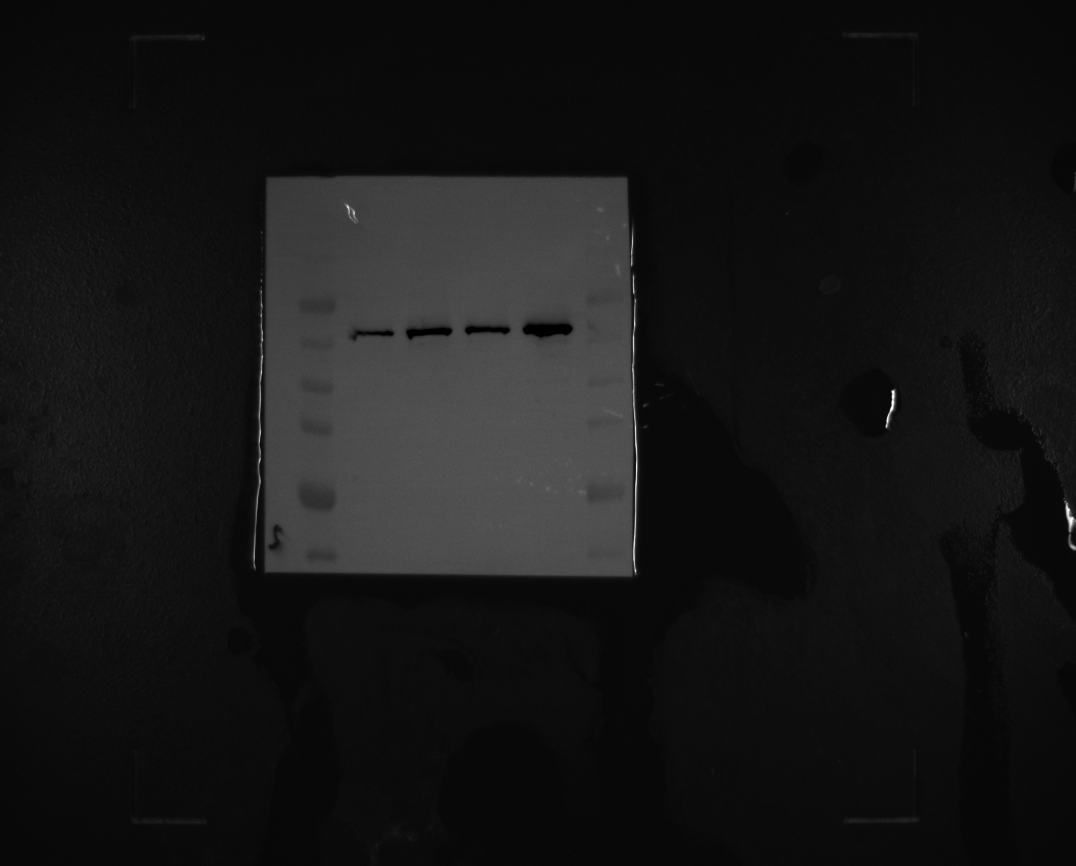


Fig.3B-P62


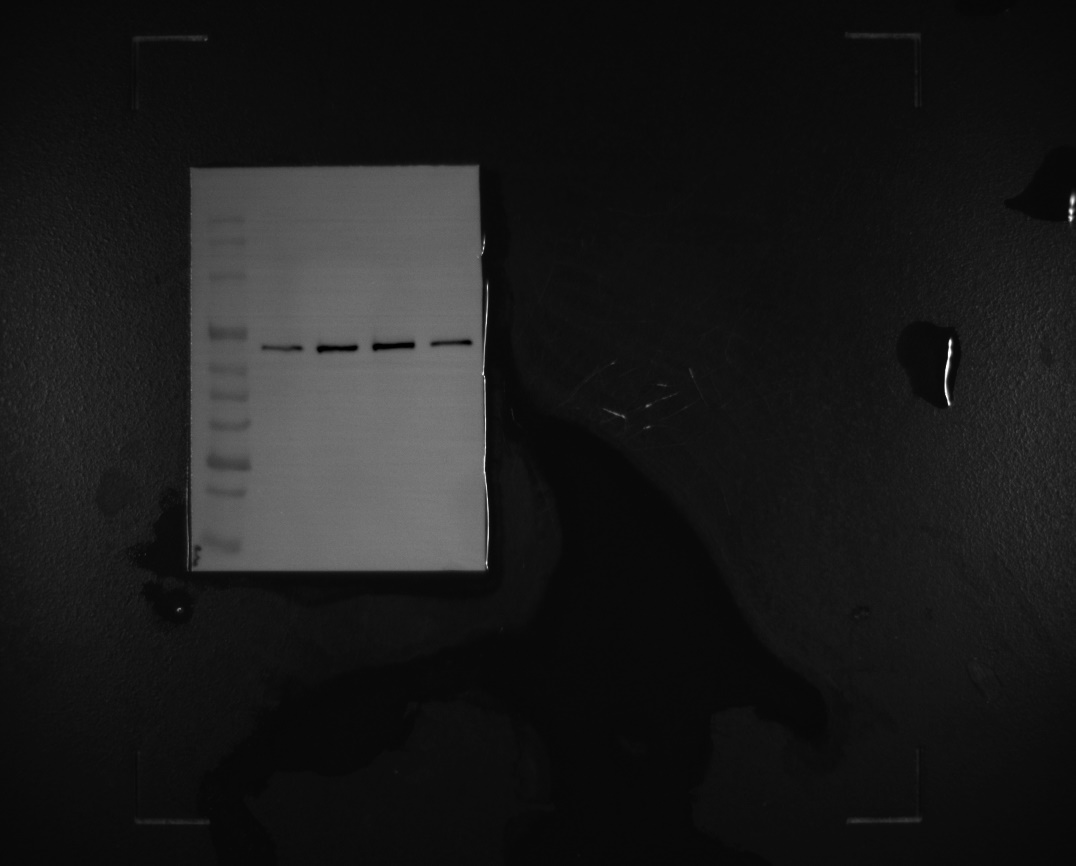


Fig.3B-β-actin


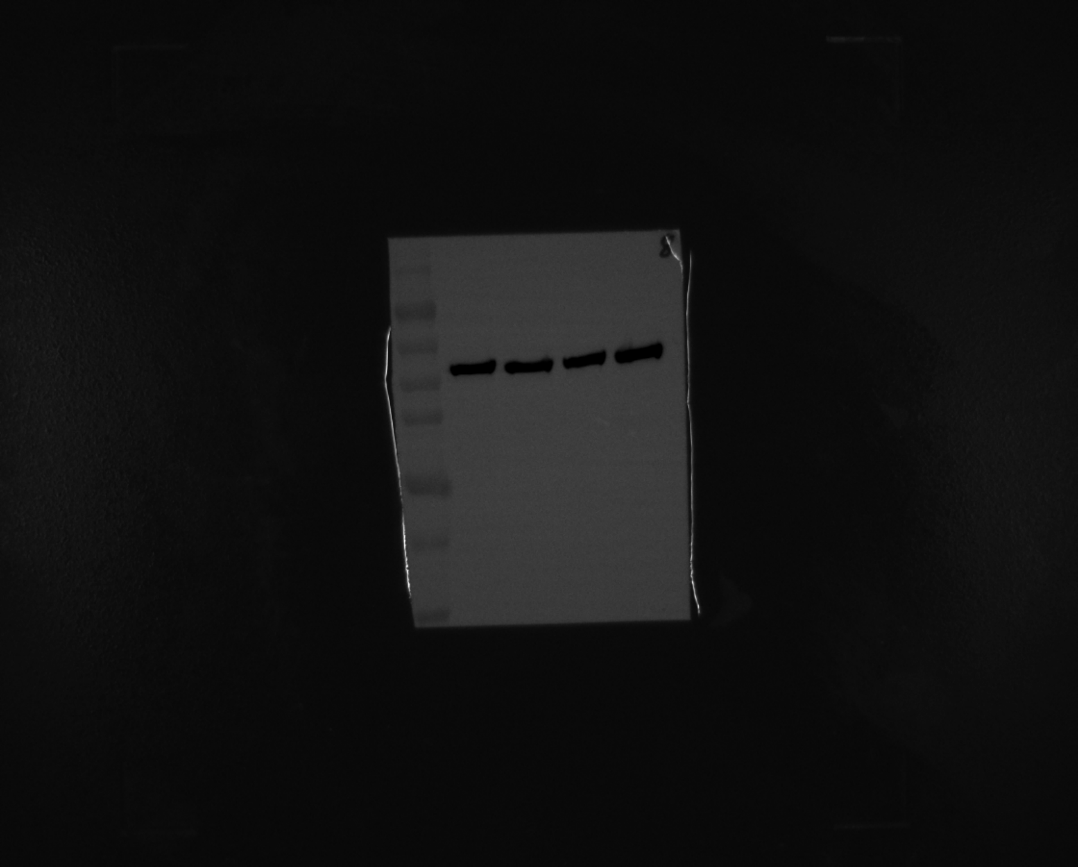


Fig.4C-P62


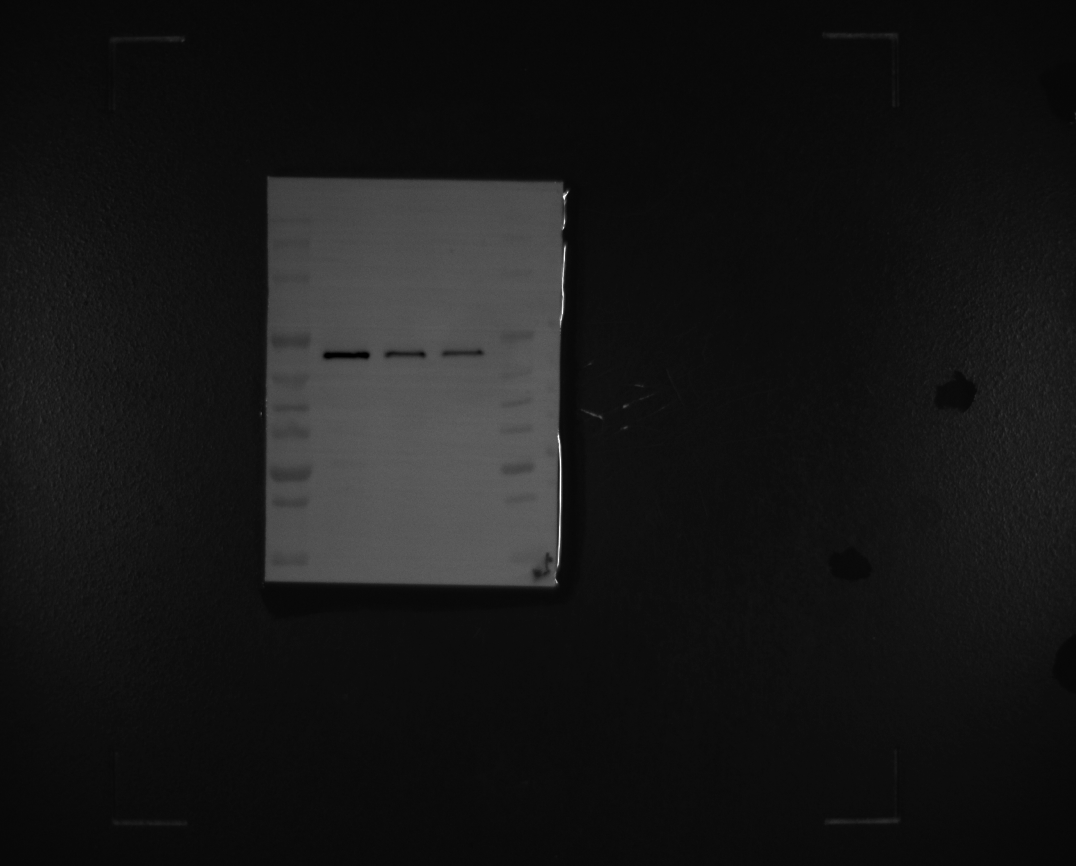


Fig.4C-LC3Ⅰ-Ⅱ


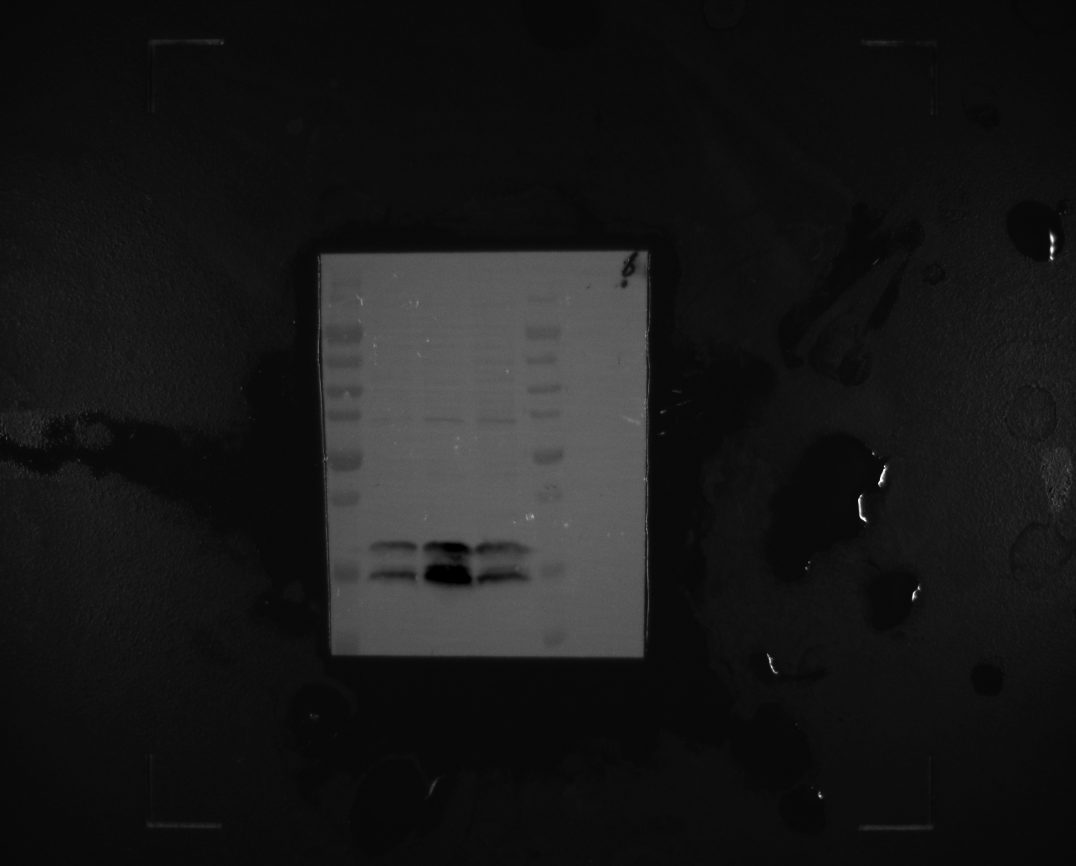


Fig.4C-Beclin-1


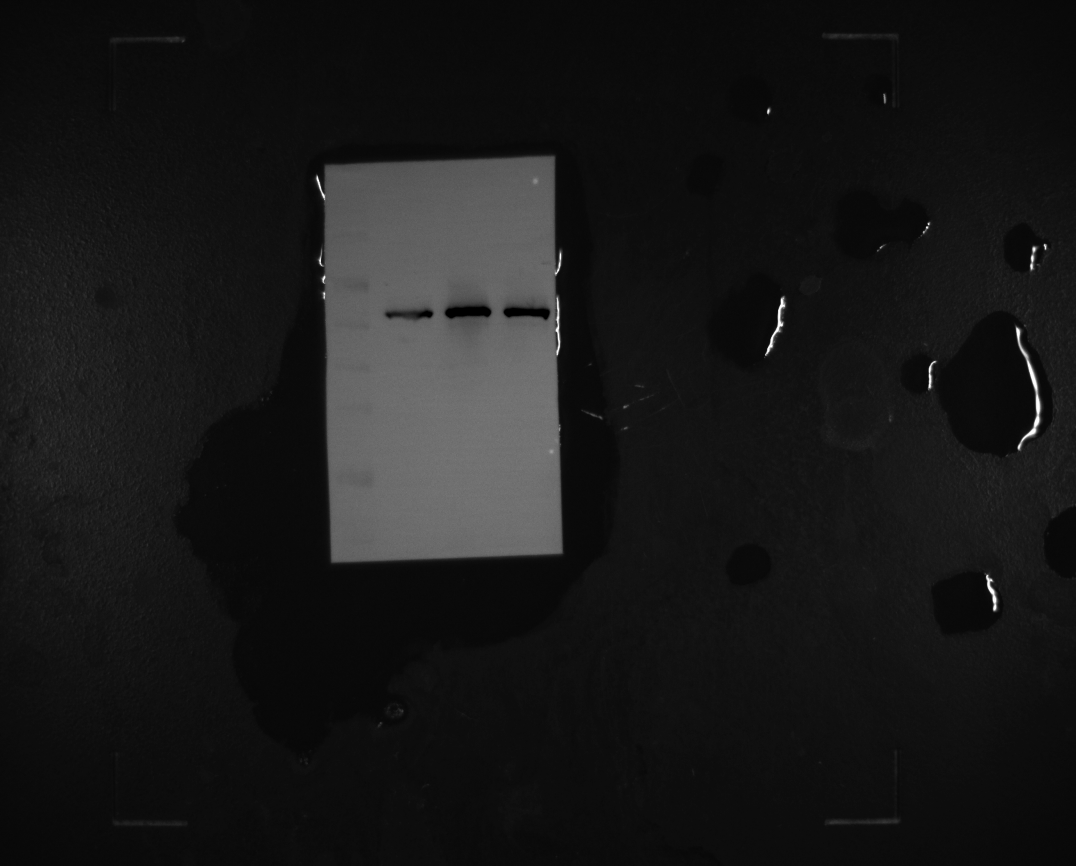


Fig.4C-β-actin


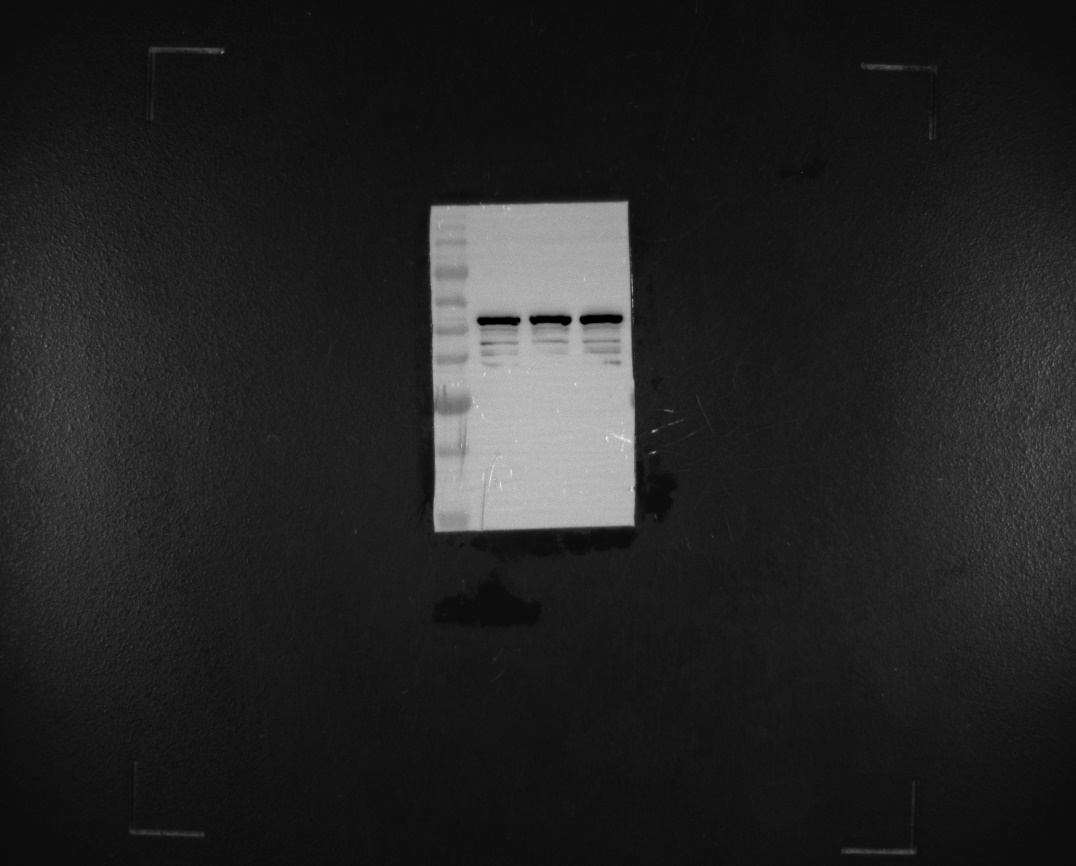


Fig.5E-PI3K


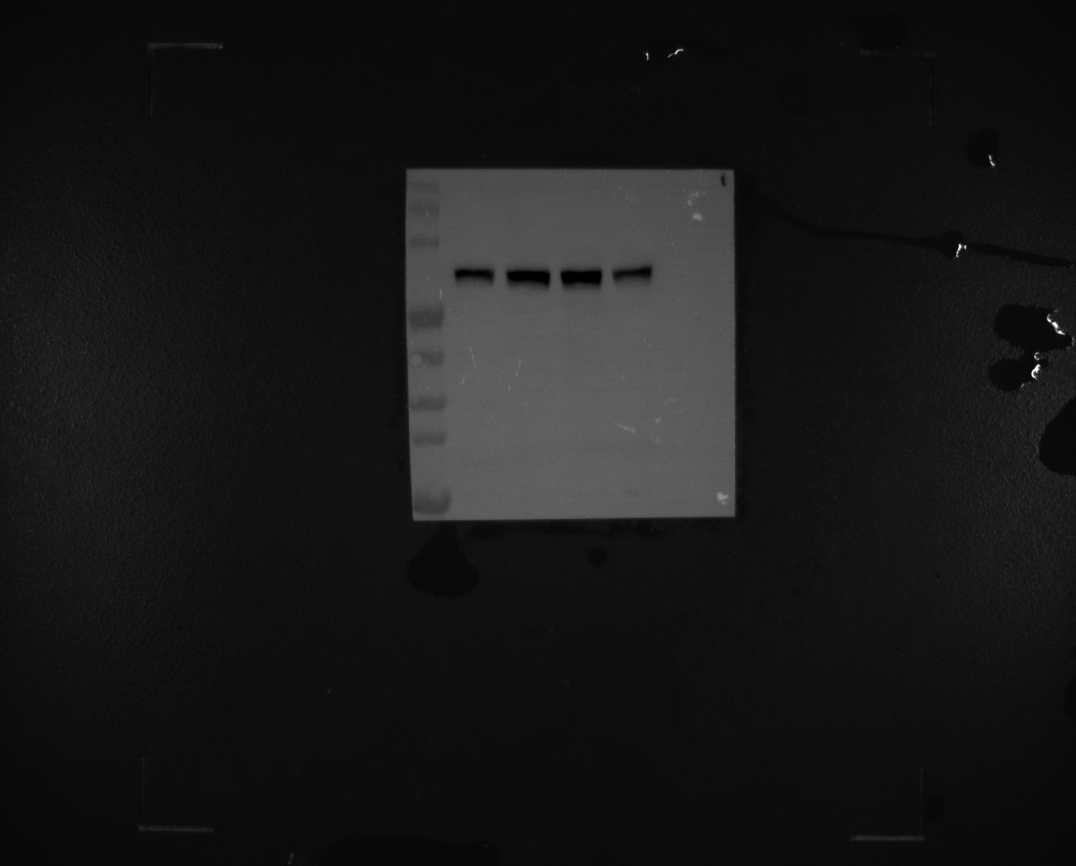


Fig.5E-p-Akt


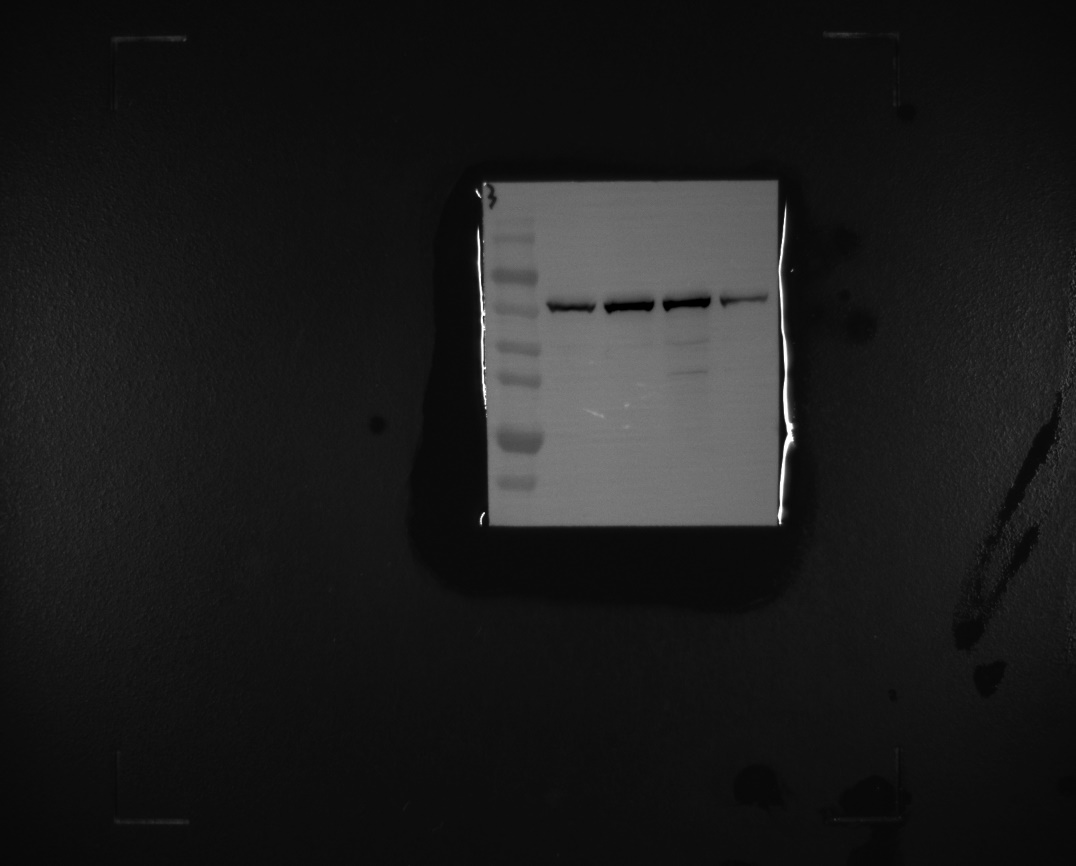


Fig.5E-Akt


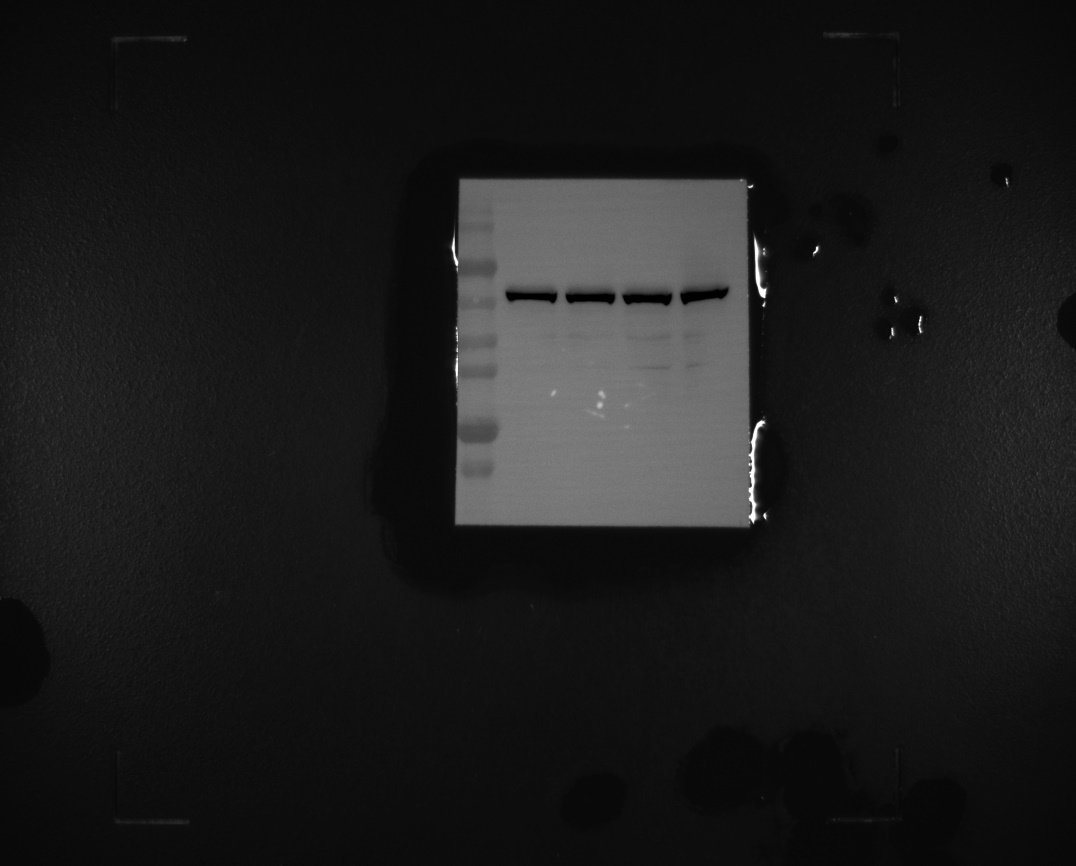


Fig.5E-p- mTOR


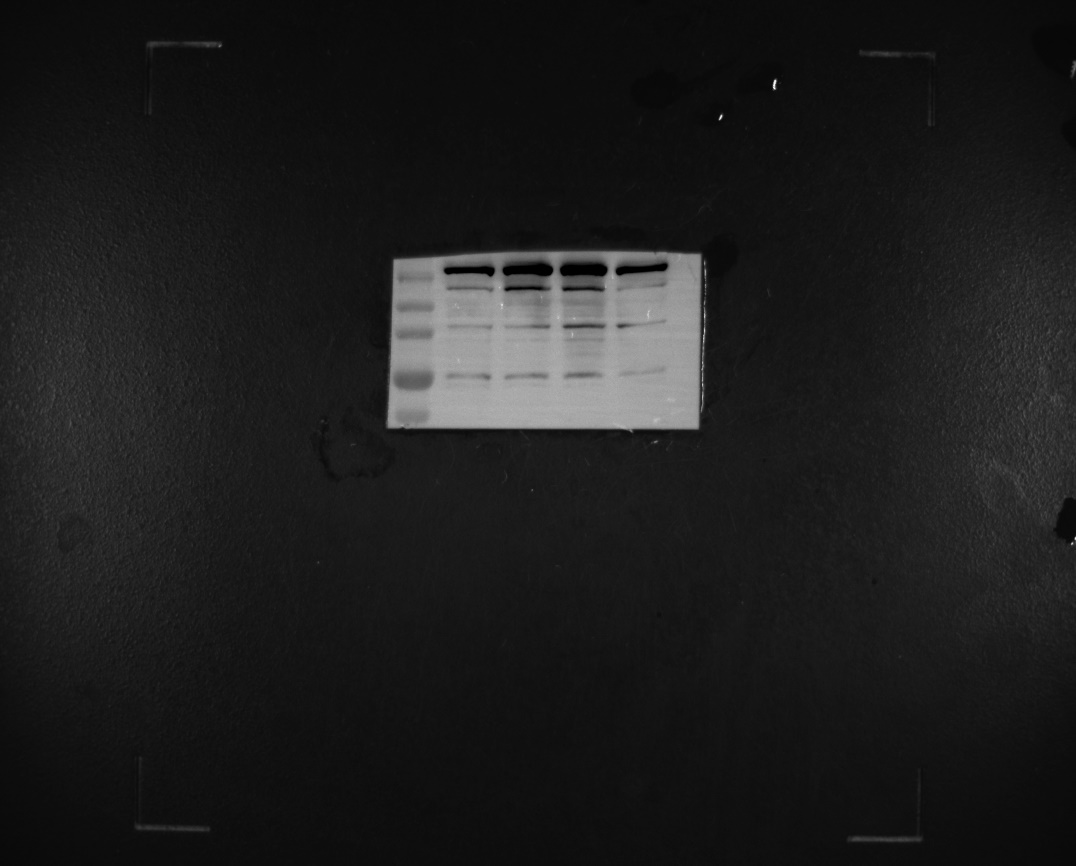


Fig.5E-mTOR


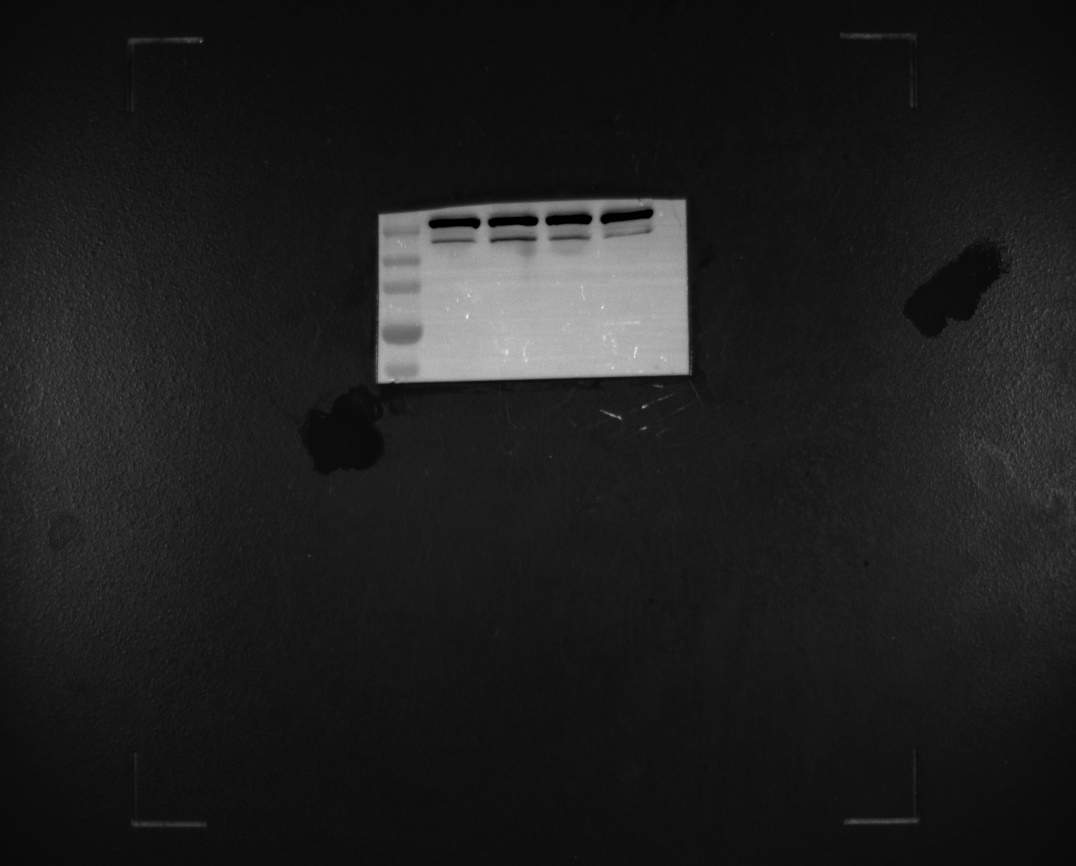


Fig.5E-α-tublin


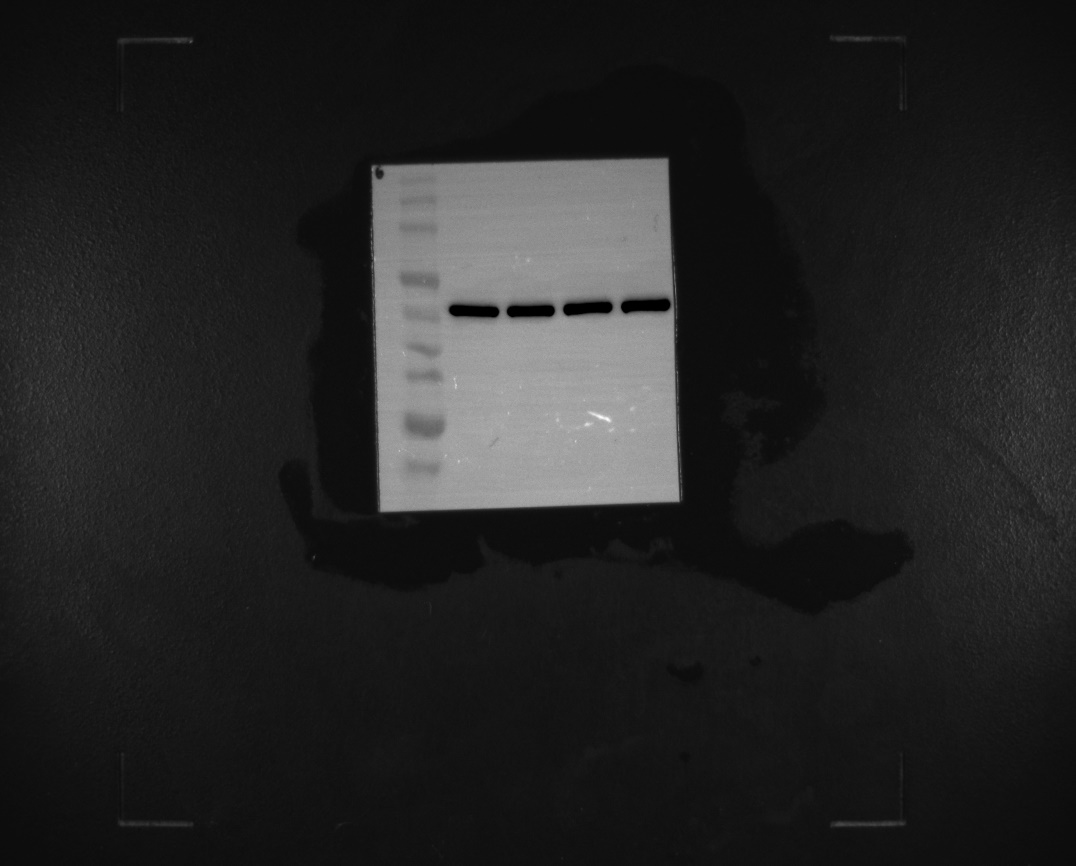


Fig.S1B-SPAG5


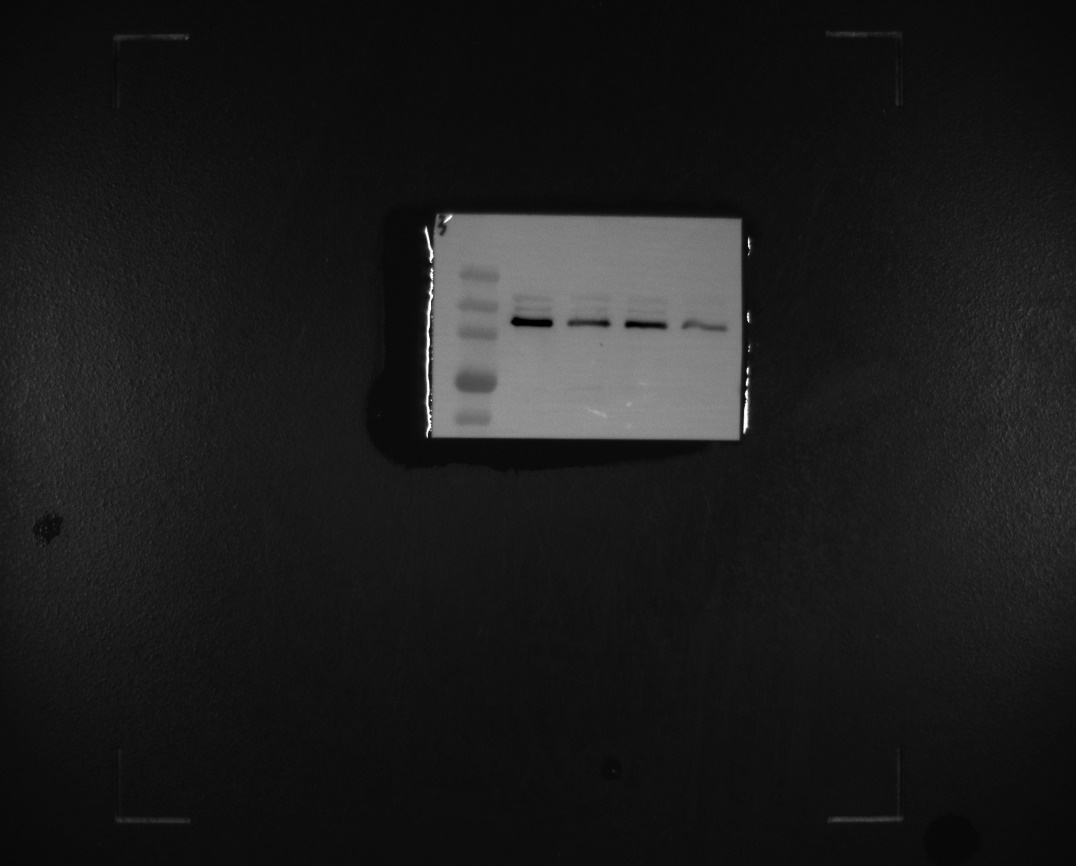


Fig.S1B-β-actin


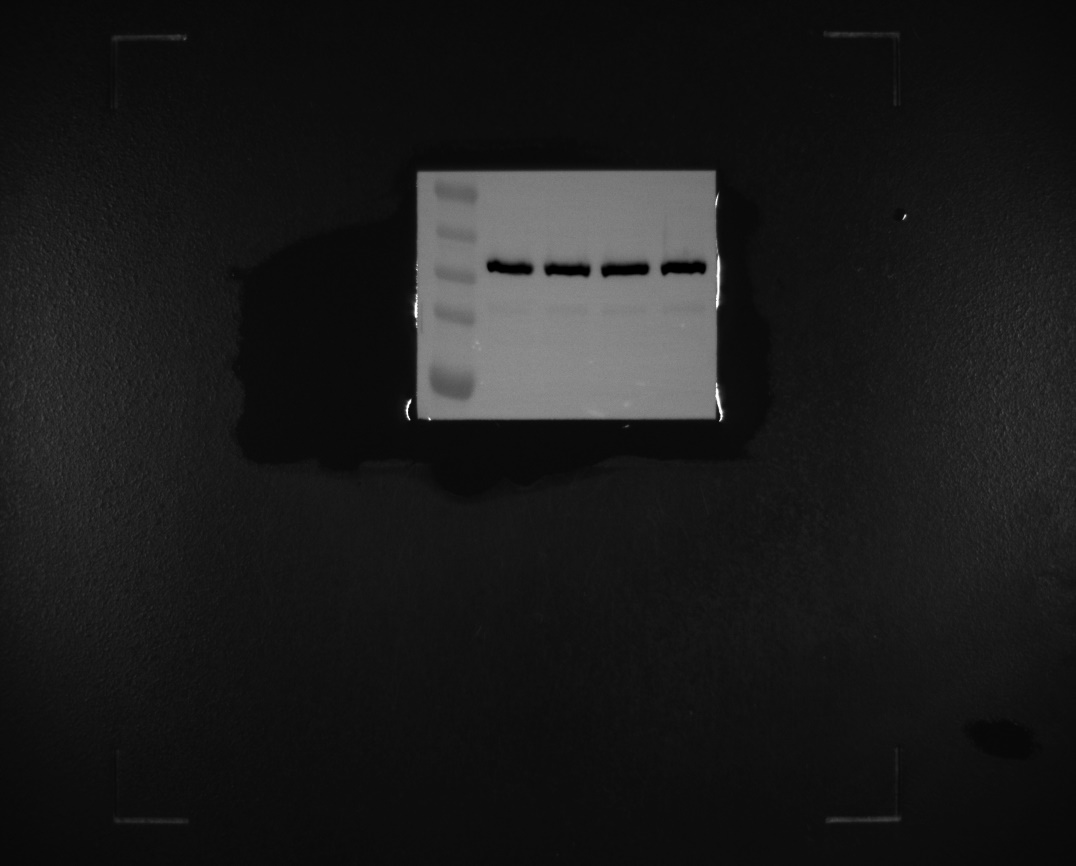


Fig.S2A- LC3Ⅰ-Ⅱ


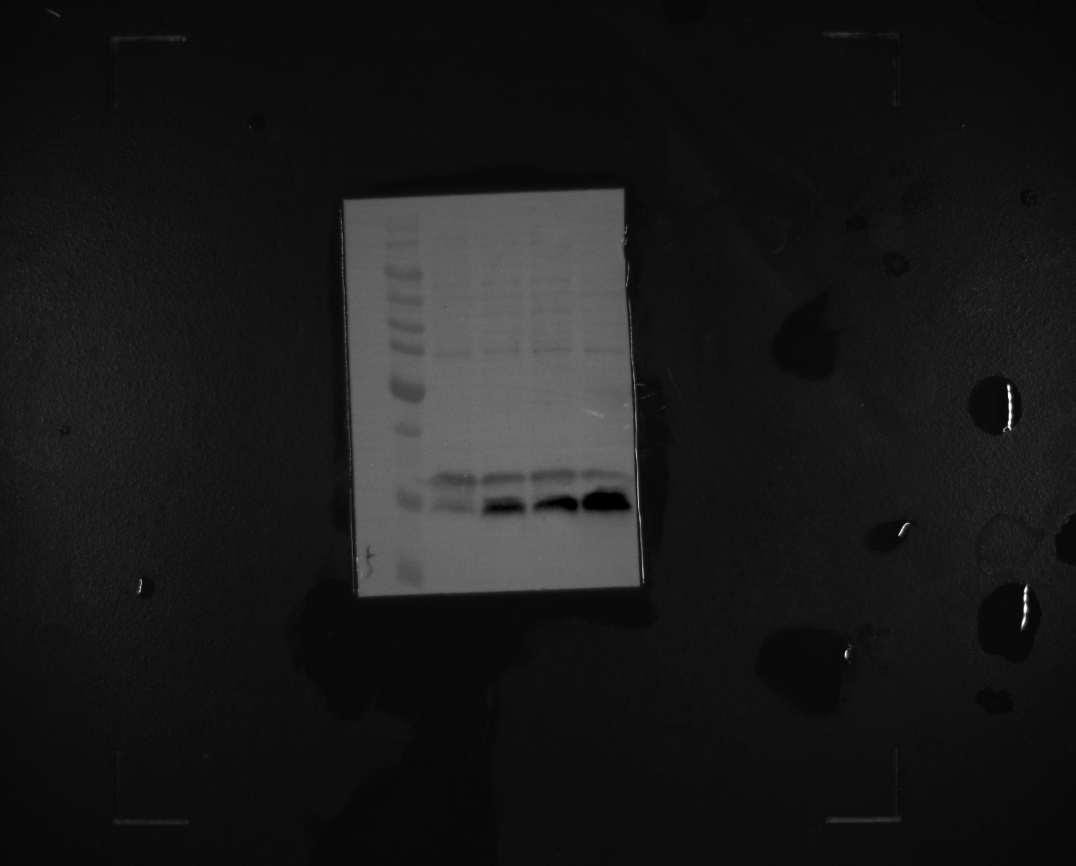


Fig.S2A-Beclin-1


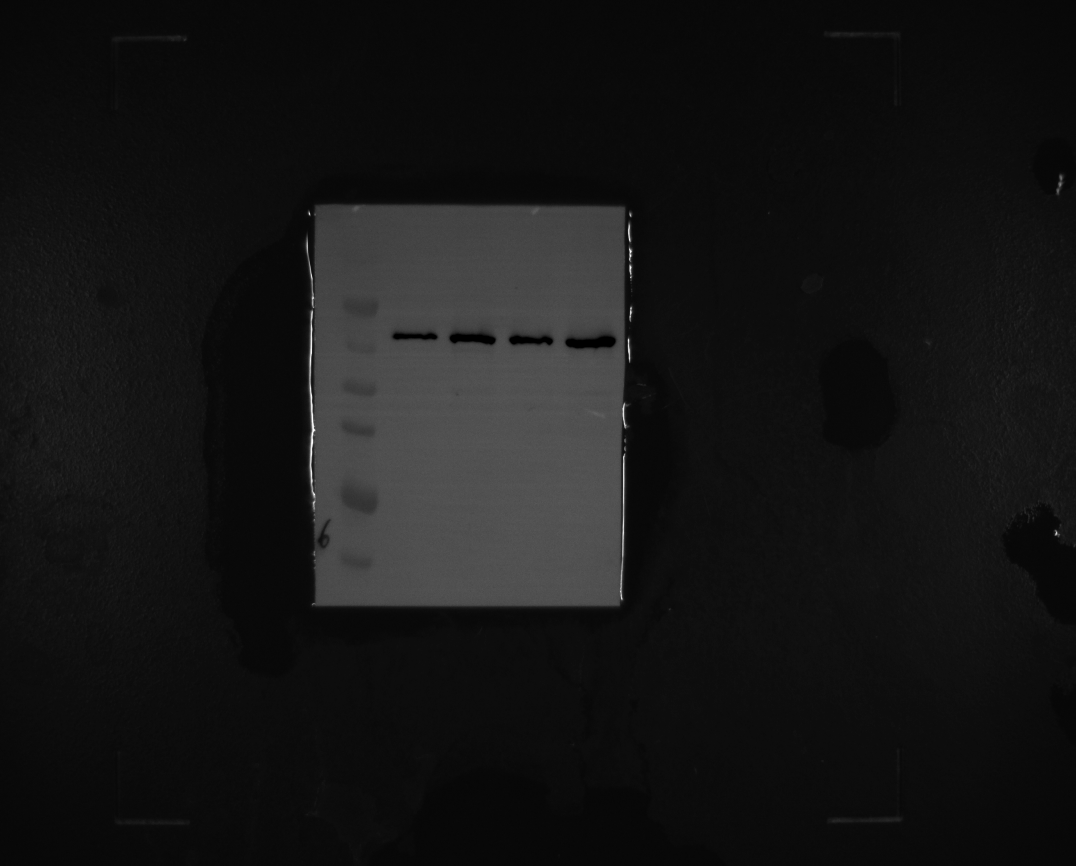


Fig.S2A-P62


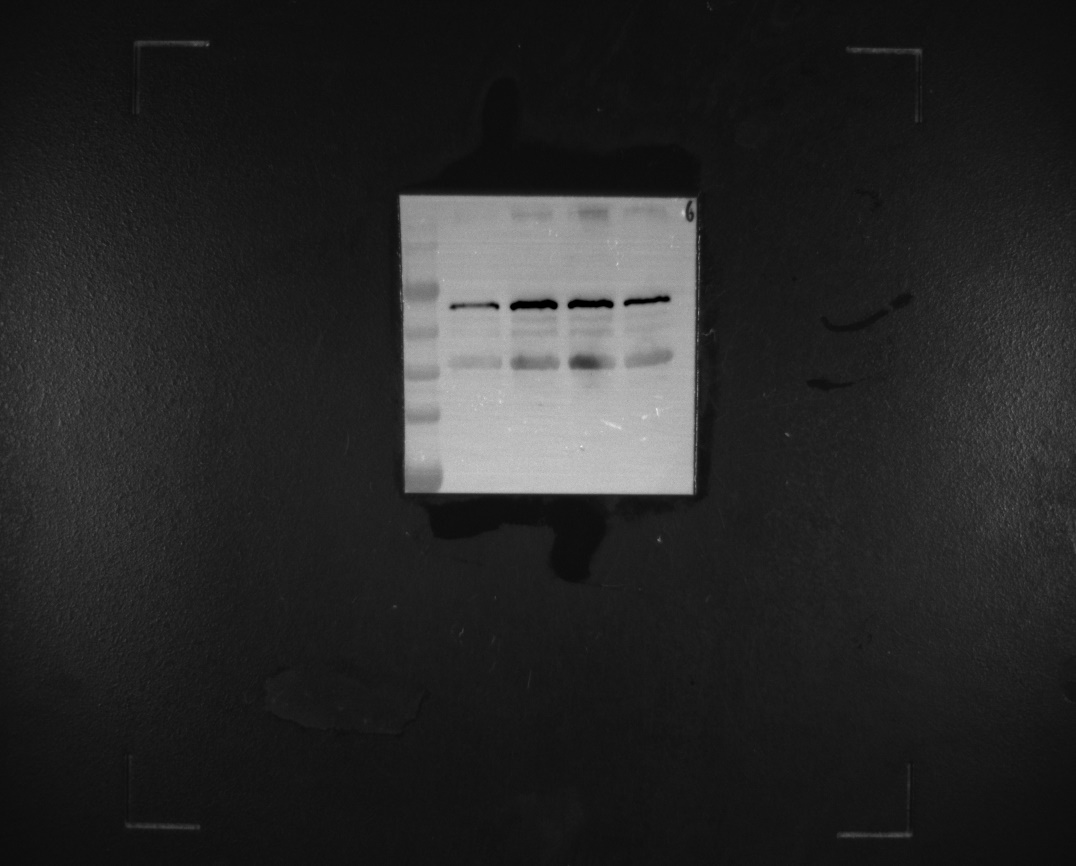


Fig.S2A-β-actin


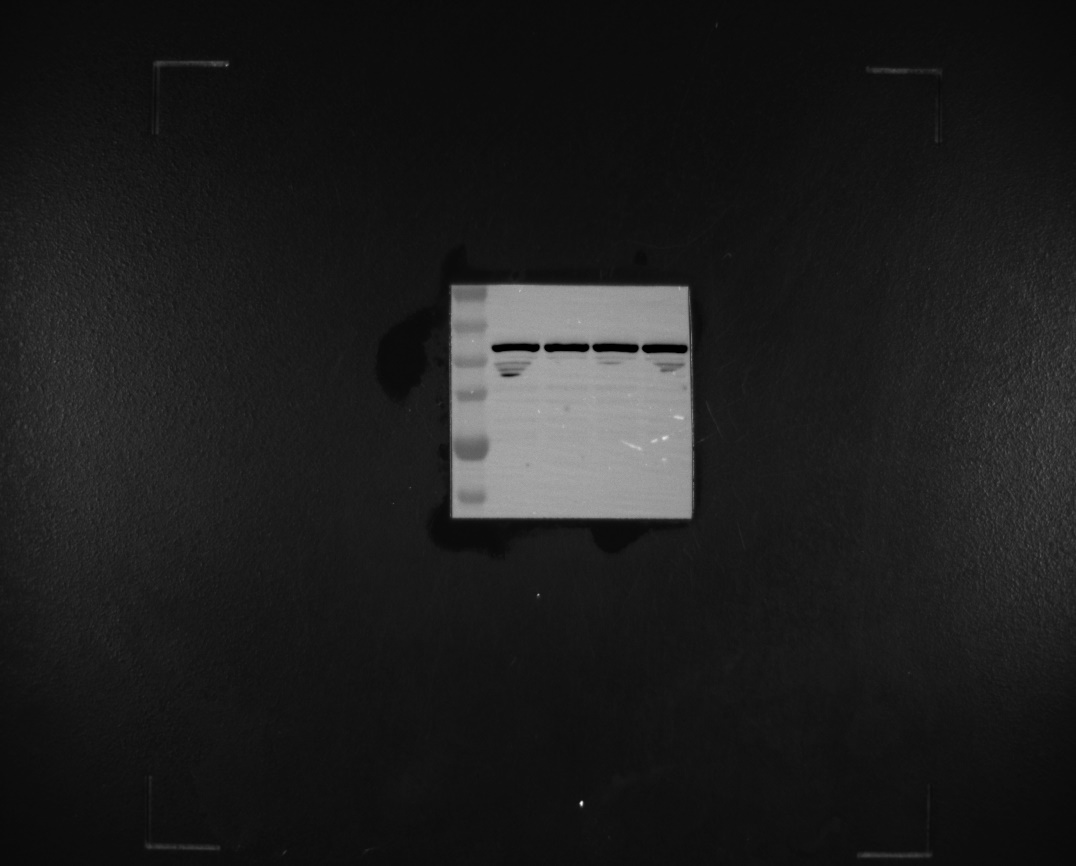

Supplement: Supplementary file 1 — Supplementary Material 1 [file 12872_2024_3945_MOESM1_ESM.docx]
