# Supplementary material for: SPAG5 deficiency activates autophagy to reduce atherosclerotic plaque formation in ApoE−/− mice
Source: BMC Cardiovasc Disord. 2024 May 28;24:275. doi: 10.1186/s12872-024-03945-5 (PMC11131316; doi:10.1186/s12872-024-03945-5)

**Fig. S1 SPAG5 knockdown reduced the expression of SPAG5 in HUVECs.**

HUVECs were transfected with si-SPAG5 or si-NC. (A) qRT-PCR examined the expression of SPAG5 in HUVECs. (B) Western blotting examined the expression of SPAG5 in HUVECs. ^*^*P* < 0.05, ^***^*P* < 0.001 vs. si-NC

**Fig. S2 SPAG5 knockdown enhanced autophagy of LPS-treated HUVECs.**

HUVECs were transfected with si-SPAG5 or si-NC, followed by 1 µg/ml LPS treatment. (A) Western blotting examined the expression of LC3-I, LC3-II, Beclin-1, p62 in HUVECs. (B) GFP-LC3 dots were detected. ^##^*P* < 0.01, ^###^*P* < 0.001 vs. Control; ^**^*P* < 0.01, ^***^*P* < 0.001 vs. LPS+si-NC.

**Fig. S1**


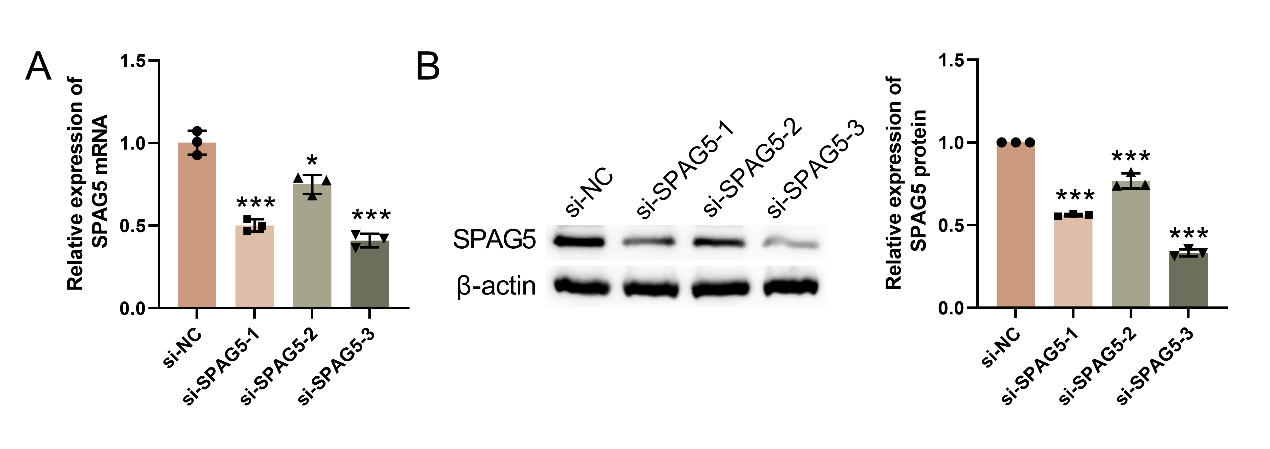


**Fig. S2**


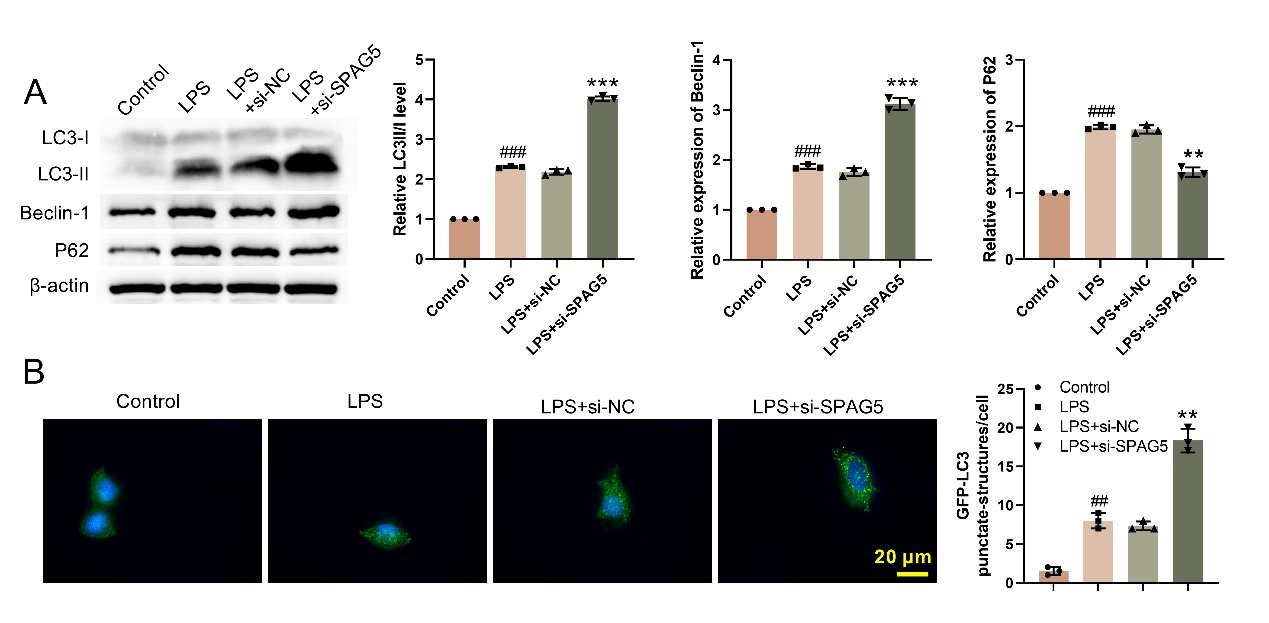

Supplement: Supplementary file 2 — Supplementary Material 2 [file 12872_2024_3945_MOESM2_ESM.docx]
